# Investigation of stress hormones across multiday seizure cycles

**DOI:** 10.1093/braincomms/fcag217

**Published:** 2026-06-08

**Authors:** Rachel E Stirling, Jodie Naim-Feil, Ian Gordon, David B Grayden, Wendyl D’Souza, Dean R Freestone, Ewan S Nurse, Mark J Cook, Philippa J Karoly

**Affiliations:** Department of Biomedical Engineering, University of Melbourne, Melbourne, VIC 3010, Australia; Graeme Clark Institute, University of Melbourne, Melbourne, VIC 3010, Australia; Department of Biomedical Engineering, University of Melbourne, Melbourne, VIC 3010, Australia; Graeme Clark Institute, University of Melbourne, Melbourne, VIC 3010, Australia; Statistical Consulting Centre, The University of Melbourne, Melbourne, VIC 3010, Australia; Department of Biomedical Engineering, University of Melbourne, Melbourne, VIC 3010, Australia; Graeme Clark Institute, University of Melbourne, Melbourne, VIC 3010, Australia; Department of Medicine, St. Vincent’s Hospital Melbourne, University of Melbourne, Melbourne, VIC 3010, Australia; Department of Medicine, St. Vincent’s Hospital Melbourne, University of Melbourne, Melbourne, VIC 3010, Australia; Department of Biomedical Engineering, University of Melbourne, Melbourne, VIC 3010, Australia; Graeme Clark Institute, University of Melbourne, Melbourne, VIC 3010, Australia; Graeme Clark Institute, University of Melbourne, Melbourne, VIC 3010, Australia; Department of Medicine, St. Vincent’s Hospital Melbourne, University of Melbourne, Melbourne, VIC 3010, Australia; Department of Biomedical Engineering, University of Melbourne, Melbourne, VIC 3010, Australia; Graeme Clark Institute, University of Melbourne, Melbourne, VIC 3010, Australia; Department of Medicine, St. Vincent’s Hospital Melbourne, University of Melbourne, Melbourne, VIC 3010, Australia; Department of Biomedical Engineering, University of Melbourne, Melbourne, VIC 3010, Australia; Graeme Clark Institute, University of Melbourne, Melbourne, VIC 3010, Australia; Department of Medicine, St. Vincent’s Hospital Melbourne, University of Melbourne, Melbourne, VIC 3010, Australia

**Keywords:** epilepsy, cortisol, multidien, infradian, epileptic rhythms

## Abstract

It is well established that most people with epilepsy experience cyclical fluctuations in seizure susceptibility. These seizure patterns have been associated with multiday oscillations in cortical excitability and autonomic changes, although the mechanistic drivers of these cycles are not well understood. In this study, we measured stress hormone levels at seizure cycle peaks and troughs (high and low risk, respectively) to investigate stress hormones as a possible co-oscillator with multiday cycles of seizure susceptibility. Thirteen participants with focal epilepsy were recruited for this longitudinal cohort study. Participants reported seizures in an electronic diary for at least 6 months prior to study commencement. Four 3-day salivary sampling periods were scheduled using a cycle forecasting algorithm trained on each participant’s historical seizure diary to prospectively identify high- and low-risk periods. Twenty-four saliva samples were collected per person across two predicted high-risk periods and two predicted low-risk periods (‘allocated risk’). Saliva samples were analysed for cortisol and dehydroepiandrosterone sulphate (DHEAS) levels. Linear mixed models were fitted to predict stress hormones with fixed effects: multiday seizure cycle (retrospective peak or trough), time of day, allocated risk, perceived stress scale score and pre- and post-sample seizure occurrence. Participants recorded an average of 47 (SD = 62) seizures between their first and final saliva collection (duration: 11.8 ± 8.0 months). Three hundred and twelve saliva samples were collected in total. Cortisol levels were significantly higher in the epilepsy cohort compared to the expected general population. On a group level, cortisol was significantly associated with fixed effects time of day, pre-sample seizure occurrence and multiday seizure cycle, with cortisol levels heightened at multiday cycle peaks compared to troughs, particularly evident in the morning saliva samples. These results provide new insights into cortisol as a possible mechanistic driver or co-oscillator of multiday seizure cycles in people with epilepsy. The novel methodology presented in this work may be used to explore interactions between other biomolecules of interest and multiday seizure cycles.

## Teaser

Stirling and Naim-Feil *et al*. report that cortisol levels are higher during peak periods of multiday seizure susceptibility in people with epilepsy. By sampling saliva at forecasted high- and low-risk times, their findings suggest that cortisol tracks underlying seizure rhythms, highlighting a potential biomarker and future target for seizure management.

## Graphical Abstract

**Graphical Abstract:**
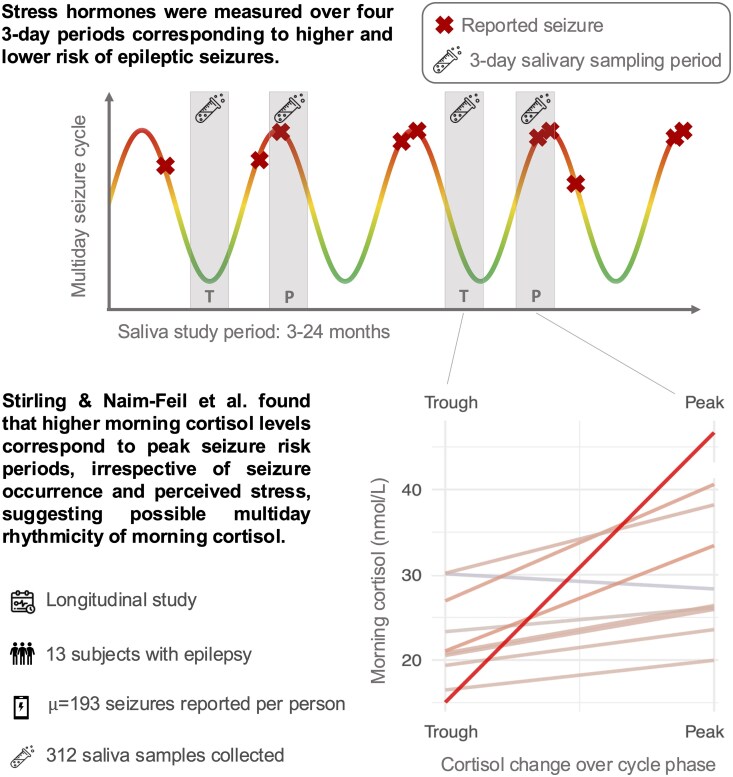

## Introduction

Epilepsy is a prevalent and debilitating neurological disorder characterized by recurrent unprovoked seizures. Various aspects of stress, both psychological and physiological, have been identified as major components of epilepsy. These include self-reported psychological stress and physiological stress responses mediated by activation of the hypothalamic–pituitary–adrenal (HPA) axis. Understanding the dynamic relationship between stress and seizure activity is challenging due to complex and bidirectional interactions between them.^1^

Psychological stress is frequently self-reported as a major trigger for epileptic seizures,^2^ with seizure diary studies linking stressful life events with increased seizure frequency.^3-6^ Acute increases in cortisol (HPA axis activation) can also precipitate epileptic seizures,^7^ while chronic stress and sustained cortisol elevation increase seizure frequency.^8^ At the same time, seizures themselves act as physiological stressors, triggering autonomic nervous system changes^8^ and activating the HPA axis,^9,10^ leading to elevated cortisol levels.^7,11^ Compared to healthy controls, basal cortisol levels are often reported to be higher in individuals with epilepsy.^12-14^ However, findings remain somewhat mixed, as other studies have reported reduced cortisol levels^15,16^ or no difference.^17,18^ Given the intricate relationship between stress and epilepsy, exploring the longer-term dynamic interplay between stress markers and seizures may enable a unifying explanation for these observations.

Epileptic seizures were long considered unpredictable events with no discernible pattern. However, recent advances in long-term intracranial EEG monitoring have revealed that both seizures and interictal epileptiform activity follow individual-specific daily and multiday (>2 day cycle) cycle patterns,^19^ occurring in about 90% of people with epilepsy.^20,21^ The discovery of rhythmic fluctuations in seizure susceptibility has pioneered a new direction in epilepsy research, with cycle-based seizure risk models consistently outperforming other seizure prediction methods.^22-25^ Notably, seizure cycles can be estimated non-invasively using self-reported seizure diaries,^22,26^ demonstrating patterns comparable to those observed in intracranial EEG recordings.^22,23,27,28^ Moreover, multiday cycles extend beyond neural activity, with similar rhythmic patterns observed in other physiological markers, such as heart rate,^29,30^ suggesting that these fluctuations may be driven by intrinsic biological processes governing multiple interconnected physiological systems, similar to circadian processes. Thus, to understand whether fluctuations in stress markers, like cortisol, align with periods of heightened seizure likelihood, it is critical to examine stress longitudinally across these individual-specific cycles. This could unlock new avenues for targeted interventions aimed at mitigating stress during high-risk seizure periods.

Cortisol levels are regulated by the body’s natural circadian rhythm, which in turn plays a significant role in maintaining metabolic homeostasis, modulating hormone responses, and influencing the sleep-wake cycle.^31,32^ Circadian fluctuations in cortisol secretion and interictal epileptiform activity appear to co-oscillate in individuals with stress-sensitive epilepsy.^33^ At slower timescales, cortisol levels appear to follow multiday rhythms, particularly seasonal and menstrual cycles in the general population.^13,34-36^ However, a very limited number of studies have explored whether these rhythms are dysregulated in epilepsy^13^ or stressed-induced.^37^ To the authors’ knowledge, no previous studies have explored how these stress markers fluctuate over multiday seizure cycles. This gap in the research exists because most existing cortisol studies are conducted over shorter timeframes and lack sufficient data to detect long-term trends (over weeks to months). Additionally, it is often infeasible or impossible to continuously monitor biomolecules (such as cortisol), over extended periods. However, novel approaches for the extraction and projection of individual-specific seizure cycles now provide a methodology capable of tracking how an individual’s stress hormones fluctuate over different phases (i.e. the peak versus the trough) of their multiday cycle, without requiring long-term, continuous monitoring. This approach may reveal previously difficult-to-obtain insights into the longer-term stress-seizure dynamics in epilepsy.

The present observational cohort study piloted a longitudinal design using cycle forecasting and modelling to examine the interaction between stress hormones [cortisol and dehydroepiandrosterone sulfate (DHEAS)], perceived psychological stress levels (self-perceived stress score) and individual-specific multiday seizure cycles. The study aimed to (i) identify whether stress markers such as cortisol, DHEAS and self-perceived stress fluctuate over multiday seizure cycles, and (ii) examine whether additional factors, such as time of day and seizure occurrence, impact the relationship between stress and seizure cycles. This novel study is the first investigation of how stress fluctuates at multiday timescales in people with epilepsy.

## Materials and methods

Eighteen adults with a clinical diagnosis of focal epilepsy were recruited from the neurology clinic at St Vincent’s Hospital, Melbourne (Australia) and from Seer Medical (Melbourne, Australia) between January 2022 and December 2024. Some of these participants were concurrently enrolled in a separate study investigating the effectiveness of scheduling diagnostic EEG sessions during high seizure risk periods.^38^

To be eligible for inclusion, participants were required to have reported at least 10 seizure events in an electronic seizure diary (Seer app, Seer Medical) over at least 6 months prior to the start of salivary sampling. Of the 18 participants initially recruited, five did not meet the inclusion criteria or were lost to follow-up due to the demands of the study protocol, including its complexity and time commitment. Clinical and demographic characteristics of participants who completed the full study protocol (*n* = 13) are provided in Table 1.

**Table 1:** Demographic and clinical characteristics of study participants

Participant	Sex	Age	Epilepsy type	Anti-seizure medications	Seizure frequency (per month) during saliva collection
P1	F	40	Focal	None	11.17
P2	M	33	Focal (RT)	Carbamazepine	1.80
P3	F	38	Focal	Lamotrigine	0.47
P4	F	36	Focal (T)	Lacosamide, Brivaracetam, Zonisamide, Losartan	6.09
P5	M	72	Focal (T)	None	0.00
P6	F	32	Focal	Levetiracetam, Lacosamide	2.77
P7	F	42	Multi-focal (T)	Zonisamide, Lamotrigine, Clonazepam, Medicinal Cannabis	4.54
P8	F	29	Focal (T)	Levetiracetam, Topiramate, Lacosamide	13.88
P9	F	31	Multi-focal	Oxcarbazepine	12.48
P10	F	25	Focal	Lamotrigine, Sodium Valproate	0.17
P11	M	38	Focal	Brivaracetam, Topiramate, Lamotrigine	3.75
P12	F	45	Focal	Lamotrigine, Levetiracetam	2.87
P13	M	47	Focal	Levetiracetam	0.12

T = temporal; RT = right temporal.

Saliva samples were collected at four predetermined time periods for each participant throughout the study to investigate changes in stress hormone levels over time. Each saliva sampling collection period lasted for 3 days and involved six saliva collections; thus, each participant provided 24 saliva samples over the study duration. Two stress-related hormones were measured in the saliva samples: cortisol and DHEAS. Salivary sampling was chosen for its non-invasive and convenient nature and is considered the gold standard for assessing free cortisol, which reflects the biologically active, unbound hormone fraction.^39^ Ethics approval for this study was granted by St Vincent’s Hospital Human Research Ethics Committee (HREC 009.19). All participants provided informed consent and were made aware that participation was voluntary, with the option to withdraw at any time.

### Study design

**Figure 1:**
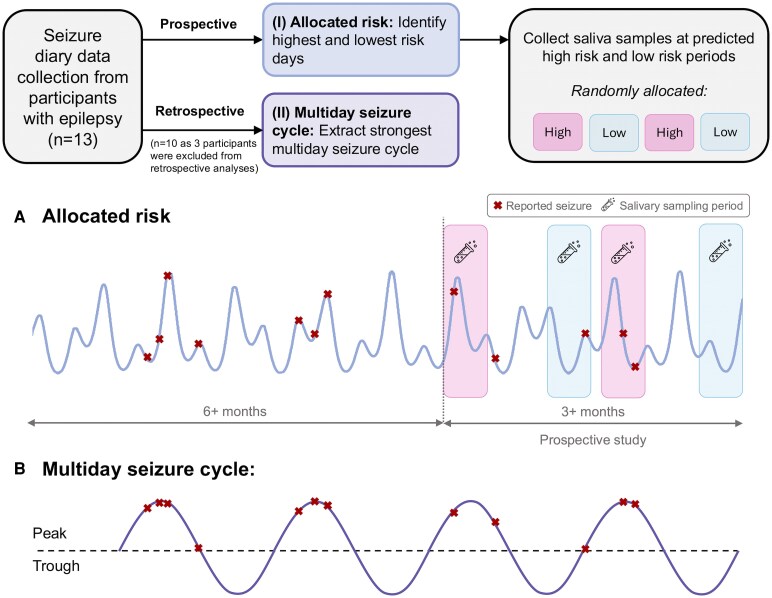
**Study protocol schematic.** Prior to salivary sampling, participants reported at least 10 seizures in an electronic seizure diary over a minimum of 6 months. Using this data, the highest and lowest risk days (two each) were identified in the next 3 months using a cycle forecasting algorithm (see ‘Allocated risk’). Participants were randomly assigned to salivary sampling during either a high-risk or low-risk period. Saliva samples were collected during two high-risk periods and two low-risk periods, each sampling round conducted over 3 days. Participants continued to use their seizure diary during the sampling phase of the study, with forecasts updated between sampling periods. At study conclusion, the seizure diary was used to extract the strongest multiday seizure cycle (retrospectively). (**A**) An example cycle forecast (generated using seizures reported before saliva sampling), which identified two predicted high-risk days (red shading) and two low-risk days (green shading), during which the saliva sampling was completed. Note that seizures (red crosses) are plotted only before saliva sampling occurred to demonstrate that only seizures reported before saliva sampling were used by the forecast to predict high- and low-risk sampling periods. (**B**) A schematic representation of a multiday seizure cycle retrospectively fitted to the whole duration of the seizure diary (i.e. seizures reported during the whole study: before and during saliva sampling). Note that the timeline presented in **A** and **B** are for illustrative purposes only; many participants had 6–36 months of seizure diary data prior to saliva sampling, with saliva sampling conducted over 3–24 months.

For data collection, this study used a cycle forecasting algorithm to extrapolate fixed sinusoidal seizure cycles, sometimes over several weeks, to schedule saliva sampling dates during high- and low-risk periods (see ‘Allocated risk’). Conversely, for data analysis, given the inaccuracy (but practicality) of the prospective approach, we estimated high- and low-risk periods using peaks and troughs of the strongest retrospective multiday cycle (see ‘Multiday seizure cycles’). A schematic of the study design and sampling protocol is shown in Fig. 1.

To schedule the dates of the four saliva sample collection periods, the cycle forecasting algorithm was used to identify the highest-risk or lowest-risk days for seizure occurrence within a 3-month window, based on each participant’s unique seizure cycles. Each saliva sample collection period involved saliva collection on three consecutive days: the day before the identified high- or low-risk day, and the two following days. This procedure was repeated across four time points (two during high-risk periods and two during low-risk periods), resulting in four 3-day sampling rounds. Participants provided six saliva samples per round (morning and evening on each of the 3 days), totalling 24 samples across the study. All participants were blinded to their risk allocation.

During each 3-day sampling round, saliva samples were collected at 8 a.m. and 8 p.m. on each day. Participants were instructed to avoid brushing their teeth or consuming caffeine, food or alcohol before the morning saliva sample and within 2 h prior to the evening sample. Participants were also asked to keep regular schedules during their sampling rounds, including consistent bed and wake times. Saliva samples were analysed for cortisol and DHEAS levels. Cortisol was measured in all six saliva samples per round to capture known diurnal fluctuations in cortisol, while DHEAS was measured only in the Day 1 morning (8 a.m.) sample, as it is more stable due to its longer half-life and minimal diurnal variation.^40^

To assess perceived stress levels, participants also completed the validated 10-item perceived stress scale (PSS)^41^ at the start of each sampling round. The PSS was used to determine whether perceived stress levels were associated with stress hormone concentrations over repeated measures,^42^ exploring the potential of the PSS as a marker of physiological stress, an association previously reported in other populations^43^ but not yet investigated in people with epilepsy. The scoring system for the PSS is reported in Supplementary Table 1.

### Allocated risk

Risk period allocation followed the methodology outlined in previous work.^38^ For each participant, a cycle forecasting algorithm was trained on the individual’s seizure diary data reported prior to the initiation of saliva sampling. Seizure diary data were processed and produced daily seizure likelihood values for 3 months into the future, which allowed us to book at least one, but often two or three, 3-day sampling periods, depending on participant availability. To ensure ongoing accuracy, the cycle forecasting algorithm was retrained 1 week before each sampling period so that the expected risk allocation reflected the most recent seizure patterns.

Seizure cycles were extracted from the seizure diary using the method presented and validated in previous work.^38,44,45^ Synchronization Index (SI; Eq. 1) values were calculated for each possible cycle period between 2 and 70 days. The SI is


(1 ) $$\mathrm{SI}(T)=\left|\frac{1}{N}\overset{N}{\underset{n=0}{\sum }}\;e^{i\theta (T,n)}\right|\;$$

where *N* is the total number of reported seizures, *n* is the seizure number in the sequence of reported seizures, θ(T,n) is the phase of the *n*th seizure relative to the cycle period *T*, and *i* is the imaginary number. The algorithm selected the strongest cycle (based on their SI values) between 2–7 days (6-h increments) and 7.5–70 days (12-h increments) to forecast risk. These parameters were chosen to align with a separate, ongoing study investigating the effectiveness of scheduling diagnostic EEG sessions during high seizure risk periods^38^ in which some participants were concurrently enrolled. These parameters have limitations (e.g. ensuring cycle periods are distinct) that may be refined in future work.

The two strongest seizure cycles were projected forward in time (beyond the last seizure diary entry) using a von Mises risk distribution model, which assumes that seizure cycles are sinusoidal in nature and have a fixed period. The von Mises risk distribution model is derived for each cycle period by mapping historic seizure times to cycle phases to produce a phase-dependent seizure likelihood distribution. Fixed seizure cycles were extrapolated beyond the seizure diary dates, and the von Mises risk distribution model was used to estimate daily seizure likelihoods. Daily seizure likelihoods for the two strongest cycles were combined using a log odds model to produce the final daily seizure likelihood forecast for 3 months into the future.

Salivary sampling round dates were chosen by selecting the highest and lowest seizure likelihood days in the next 3 months and then repeating this process until all sampling rounds were completed. Each sampling round lasted for 3 days and was conducted around the highest or lowest risk date (day before, day of, day after). The order in which these risk states were completed was random, based on participant availability and convenience.

### Multiday seizure cycles

After the study was completed and all salivary samples were collected, the strongest seizure cycle was *retrospectively* modelled from the complete seizure diary, using seizure events reported prior to, between and during salivary sampling periods. Incorporating all reported seizure events strengthens the accuracy of the multiday seizure cycle model, as the prospective *Allocated Risk* forecast relies on the assumption that biological rhythms can be projected using fixed period models, which does not appear to hold true in real-world biological systems.

To determine the strongest seizure cycle, the SI was calculated on all possible cycle periods between 4 and 200 days, in increments of 12 h. The strongest seizure cycle (i.e. highest SI value) was fitted to the seizure diary using a fixed period, repeating von Mises risk distribution model, whereby cycle peaks represent highest seizure risk days and troughs represent lowest seizure risk days. Once the strongest multiday cycle was modelled, salivary sampling periods were categorized as occurring during either a cycle peak or a cycle trough. This classification was based on the cycle phase on the second (middle) salivary sampling day, with peaks and troughs defined as days landing above or below the 50th percentile of all cycle likelihoods, retrospectively (Fig. 1). The binary classification (peak, trough) of multiday seizure cycle phase was ideal to mirror our peak/trough sampling design, remain consistent with prior work^38^ and mitigate diary-based phase uncertainty, while avoiding the added complexity of circular parameterization required for continuous phase; with larger samples, this approach could be expanded to multiple phases or continuous circular modelling.

### Statistical analysis

#### Linear mixed models

Linear mixed models (with Nelder-Mead optimizers) were fitted to predict log-transformed concentrations of the biomolecules of interest (Eq. 2): cortisol concentration (nmol/L), DHEAS concentration (nmol/L) and the derived cortisol:DHEAS ratio. Each model included the following variables as fixed effects: time of day (morning versus evening), allocated risk period (high versus low), pre-sample seizure, post-sample seizure, multiday seizure cycle (peak versus trough) and PSS score. The model included saliva sampling collection period (i.e. 1, 2, 3, or 4) as a random effect, nested under each participant,


(2 ) $$\log\;(Y_{i})\;∼\;\beta_{0}+\beta_{\mathrm{time}}X_{\;\mathrm{time}\;}+\beta_{\mathrm{risk}}X_{\mathrm{risk}}+\beta_{\mathrm{cycle}}X_{\mathrm{cycle}}+\beta_{\mathrm{pre}\text{-}\mathrm{seizure}}X_{\mathrm{pre}\text{-}\mathrm{seizure}}+\beta_{\mathrm{post}\text{-}\mathrm{seizure}}X_{\mathrm{post}\text{-}\mathrm{seizure}}+\;\beta_{\mathrm{PSS}}X_{\mathrm{PSS}}+(1\;|\;\mathrm{sampling}\;\mathrm{period}:\mathrm{participant})+\;\varepsilon$$

where $Y_{i}$ denotes the concentration of the biomolecule of interest, $\beta_{0}$ is the fixed intercept, ε is the error, $\beta_{i}$ are the fixed effect coefficients and $X_{i}$ are the fixed effect variables representing:

- **Time of day (** $X_{time}$ **):** whether the saliva sample was provided in the morning (0) or night (1).
- **Allocated risk (** $X_{risk}$ **):** whether the saliva sample was given during an allocated high (1) or low (0) risk period. The sampling date was scheduled up to 3 months in advance and confirmed or adjusted 1 week prior, following retraining of the forecasting algorithm on the participant’s most recent seizure data. This is similar to intention-to-treat analysis in clinical trials and evaluates the real-world effectiveness of measuring biomolecules at assigned risk states.
- **Multiday seizure cycle (** $X_{cycle}$ **):** whether the salivary sampling period was conducted at a retrospective multiday seizure cycle peak (1) or trough (0). This is similar to the actual treatment received variable in clinical trials and captures the actual cycle phase at which a biomolecule was measured.
- **Pre-sample seizure (** $X_{pre\text{-}seizure}$ **):** whether (1) or not (0) a seizure was reported within 12 h *before* the saliva sample was provided, as post-ictal changes in cortisol have previously been reported, particularly for generalized tonic-clonic and focal impaired awareness seizures.^46^
- **Post-sample seizure (** $X_{post\text{-}seizure}$ **):** whether (1) or not (0) a seizure was reported within 12 h *after* the saliva sample was provided, as pre-ictal changes in cortisol have previously been reported.^47^
- **PSS score (** $X_{PSS}$ **):** total PSS score, where higher values indicate higher perceived stress levels (Supplementary Table 1). This fixed effect was scaled per 10 units (i.e. divided by 10) to ensure confidence intervals were comparable to other variables.

Cortisol, DHEAS and cortisol:DHEAS ratio were log-transformed prior to model fitting to reduce the effect of skewed data. Models were fitted using the lme4 package in R, with random intercepts specified for each participant and saliva sampling collection period to account for repeated measures. Fixed effects significance was assessed using ANOVA with Kenward–Roger correction for adjusting degrees of freedom.

In addition to the primary model (Eq. 2), we conducted a series of supplementary linear mixed models to examine the influence of other variables. These variables were as follows. (i) An alternative pre- and post-sample seizure window, in which seizure occurrence within 1 h before or after saliva collection was modelled instead of the ±12 h window used in the main analysis. This variation aligns with pre- and post-ictal windows used in previous analyses, although it was not preferred in the primary analysis because of the small number of seizures that were reported within the ±1 h sample window; (ii) *Days Since Last Seizure* as an additional fixed effect, to capture temporal dynamics of seizure timing not otherwise accounted for by $X_{\mathrm{cycle}}$, $X_{\mathrm{pre}\text{-}\mathrm{seizure}}$ and $X_{\mathrm{post}\text{-}\mathrm{seizure}}$; and (iii) *Time Since Waking* as an additional fixed effect, to account for the diurnal variation in cortisol beyond time of day.^48^ Time Since Waking was extracted for participants wearing a smartwatch (Fitbit, Alphabet Inc.). Sleep data were provided by Fitbit using a proprietary sleep algorithm, which estimates sleep onset and offset using accelerometry and photoplethysmography. Although informative, the *Time Since Waking* variable was not included in the primary model because it was not available for all participants and reduced the total number of observations by approximately 25%.

#### Multiday seizure cycle and cortisol

Sub-analyses were conducted to further understand the relationships observed in the linear mixed models. First, the strength of the association between cycle phase (i.e. *Multiday seizure cycle* variable) and cortisol concentration was assessed with two permutation tests: (i) Shuffling of the observed peak/trough labels across the four sampling rounds within each participant, which preserves participants’ original proportions of peak rounds to trough rounds while breaking any systematic alignment between cortisol and cycle phase, and (ii) Independent random reassignment of peak/trough labels for each sampling round per participant. For each approach (*n* = 2000 permutations), we refitted the cortisol linear mixed model and extracted the *Multiday seizure cycle* coefficient to generate a null distribution.

Second, an additional linear mixed model was fitted to predict the effect of multiday cycle phase on all morning and evening cortisol samples. The model included $X_{\mathrm{cycle}}$ as a random effect, nested under each participant, allowing individual-specific baseline cortisol levels and cycle phase-related change in cortisol to be accounted for:


(3 ) $$\log\;(Y_{i})\;∼\;\beta_{0}+\beta_{\mathrm{cycle}}X_{\mathrm{cycle}}+(\;X_{\mathrm{cycle}}|\;\mathrm{participant})+\;\varepsilon$$

where $Y_{i}$ denotes the concentration of morning or evening cortisol. Slopes of the random effects (i.e. the individual relationships between cortisol concentration and multiday cycle phase) were calculated, with the significance of each slope tested using a two-sided *t*-test on a simple linear regression model of log cortisol (morning or evening) with multiday cycle phase as the predictor (two-sided *t*-test on the slope).

Third, to explore factors contributing to different slopes, we constructed linear models predicting the slope of cortisol change across multiday cycles, stratified by time of day (morning versus evening samples). Stepwise regression using backward selection was performed to iteratively remove non-informative predictors based on Akaike Information Criterion. Separate models were fitted for morning and evening samples using predictors: intra-individual variability in cortisol and DHEAS levels, multiday cycle strength (i.e. SI), circadian cycle strength and circadian seizure chronotype (mean phase of seizure timing on circadian clock, where 1 = morning phase, i.e. 12 a.m. to 12 p.m., and 0 = afternoon phase, i.e. 12 p.m. to 12 a.m.).

Fourth, given the expected confounding impact of seizure occurrence on stress hormone concentrations, further investigations into pre- and post-sample seizure occurrence were conducted. Standardized cortisol and DHEAS concentrations were compared between samples that were given at times when no seizure occurred in close proximity (no seizure samples) and samples that were given within 1-h or 12-h *before* or *after* a seizure (*pre-* and *post-*sample variables, respectively). Group differences were assessed using Mann–Whitney–Wilcoxon tests, with Bonferroni correction applied to correct for multiple comparisons (corrected significance threshold: *P* < 0.0083).

#### Other analyses

Differences in the distributions of stress hormone levels across the population at multiday cycle phase and time of day were also tested, using the Kolmogorov–Smirnov test and *P*-values were reported. The Pearson’s correlation test was used to measure the correlation between items on the PSS scale and hormone levels and seizure frequency and hormone levels. Any other statistical tests used in the supplement are reported in their respective figure or table captions.

All statistical analyses were conducted in R (version 4.4.0).

## Results

Thirteen participants with epilepsy completed the study (see Table 1 for demographic and clinical characteristics). Participants recorded a mean of 146 (standard deviation (SD) = 158) seizures over 29.3 months (SD = 31.8) prior to beginning saliva sampling, and a mean of 47 (SD = 62) seizures between their first saliva collection and their final saliva collection (duration mean ± SD: 11.8 ± 8.0 months). All participants provided 24 saliva samples (12 morning and 12 night) throughout the study. Participants’ mean morning cortisol, evening cortisol and DHEAS concentrations were 33.7 nmol/L (SD = 11.5 nmol/L), 6.4 nmol/L (SD = 2.7 nmol/L) and 14.9 nmol/L (SD = 10.3 nmol/L), respectively. While this study did not explicitly verify the full circadian profile of cortisol, all participants exhibited the expected diurnal pattern, with higher morning than evening cortisol levels. DHEAS levels across the cohort were not significantly different from the expected general population (Supplementary Fig. 1). In contrast, participants had substantially higher cortisol levels compared to the expected general population (*P* < 0.001), regardless of whether the sample was provided at a multiday seizure cycle peak or trough (Supplementary Fig. 2) or in the morning or evening (Supplementary Fig. 3).

Of the participants who completed the study, three participants with insufficient diary reported seizures (P5 and P13) or unusually long multiday seizure cycles (P3) were excluded from retrospective multiday seizure cycle analyses, to avoid biased inference arising from unreliable cycle phase assignment (Supplementary Fig. 4), resulting in a cohort of ten participants. The following results are from the cohort of participants included in the final analysis (*n* = 10).

**Figure 2:**
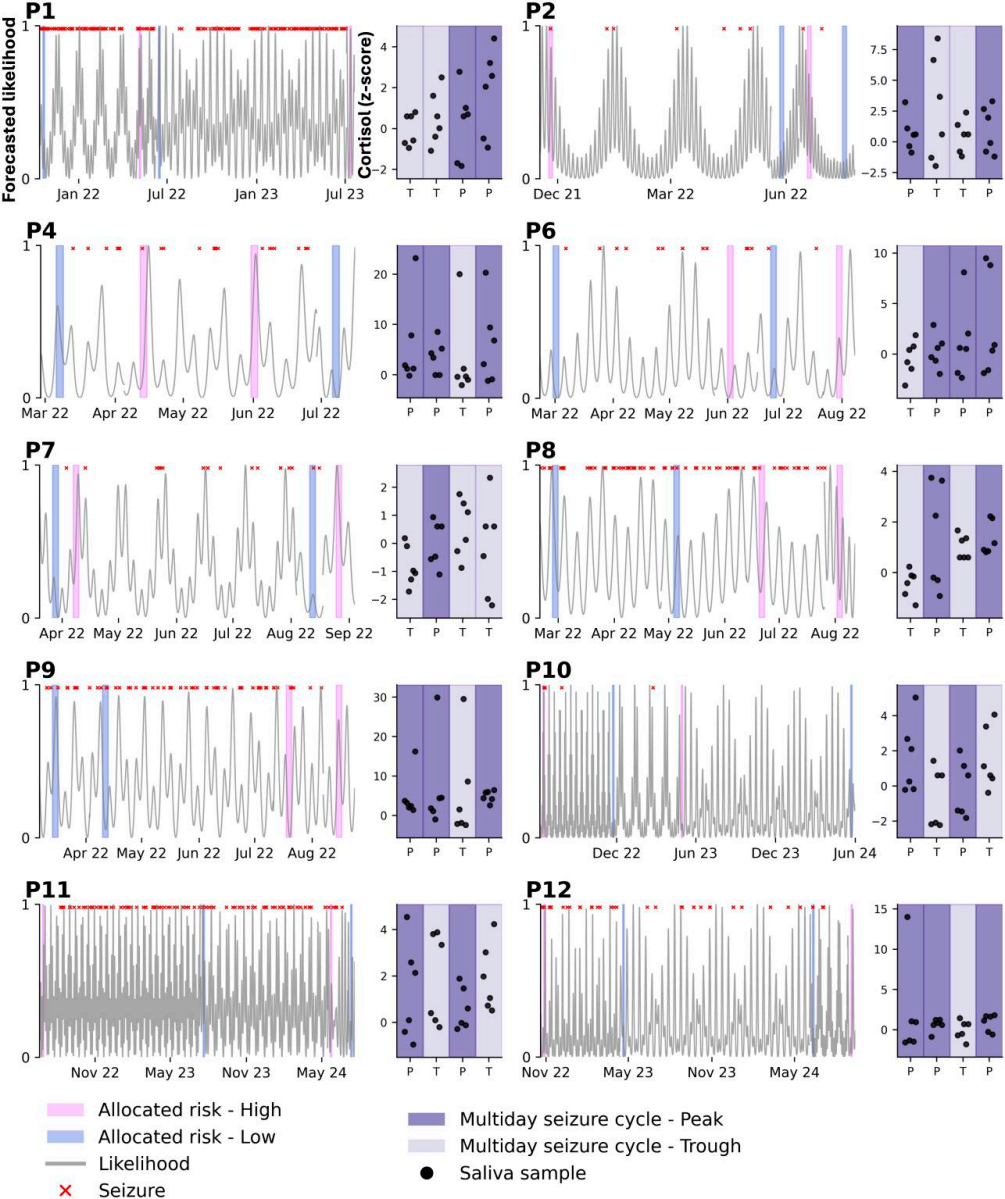
**Prospective cycle forecast and allocated risk (left panels), and corresponding multiday seizure cycle phase with standardized cortisol levels (right panels).** Only participants included in the retrospective analyses are shown (*n* = 10, participants P1, P2, P4, P6, P7, P8, P9, P10, P11 and P12). For each participant, the left panel shows the prospective likelihood from the cycle forecast (grey line), with reported seizures overlaid at the top border (red crosses). Shaded regions indicate the scheduled sampling periods, coloured by allocated risk (pink: high risk; blue: low risk). The right panel displays standardized cortisol concentrations (*z*-scores; black markers) measured during the scheduled sampling periods (*n* = 24 per participant). Background shading in the right panel represents the retrospective multiday seizure cycle phase during each sampling window [dark purple: cycle peak (P); Light purple: cycle trough (T)]. In both panels, shaded regions correspond to the same sampling periods, enabling direct visual comparison between allocated risk, multiday seizure cycle and observed cortisol dynamics.

While not all allocated risk states were perfectly aligned with retrospective cycle phase (Fig. 2), a mixed-effects logistic regression with random intercepts for participant revealed that being allocated into the high-risk state was associated with 3.1-fold higher odds of being at a multiday seizure cycle peak (OR = 3.1, 95% CI: 1.8–5.4, *P* < 0.001). Participants had a mean SI of 0.35 (SD = 0.20) of reported seizure events with respect to their multiday seizure cycle (Supplementary Table 2).

### Linear mixed models

**Figure 3:**
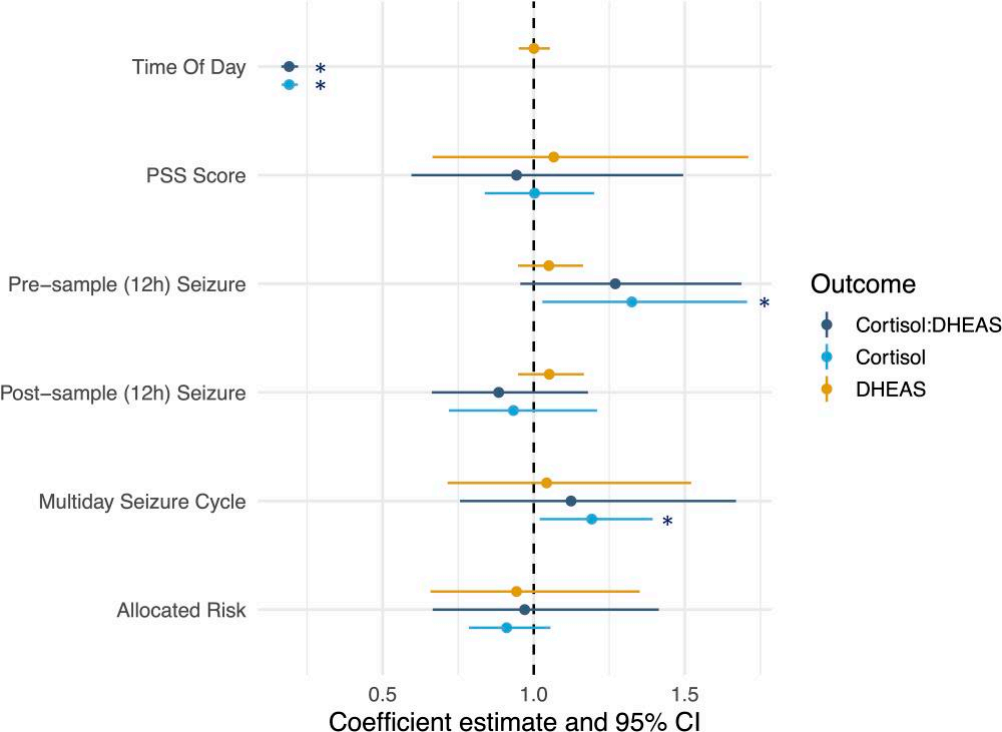
**Coefficient estimates and confidence intervals for fixed effects in the linear mixed models (*n* = 240 observations).** Three models were evaluated and plotted together: cortisol concentration (blue), dehydroepiandrosterone sulphate (DHEAS) concentration (yellow) and cortisol:DHEAS ratio (dark blue) (Eq. 2). Coefficient estimates and 95% confidence intervals (CIs) were exponentiated, giving a ratio measure of effect on the response variables. *Indicates significance of the coefficient (*P* < 0.05), derived from Wald *t*-tests. Wald *t*-statistic (*P*-value) for each significant coefficient: cortisol/time of day = −22.36 (*P* < 0.001); cortisol:DHEAS/time of day = −22.50 (*P* < 0.001); cortisol/pre-sample (12 h) seizure = 2.19 (*P* = 0.030); cortisol/multiday seizure cycle = 2.30 (*P* = 0.029).

Linear mixed models (Eq. 2) were fitted to predict log-transformed cortisol concentration, cortisol:DHEAS ratio and DHEAS concentration using the fixed effects of time of day, multiday seizure cycle, allocated risk, pre-sample seizure, post-sample-seizure and PSS score, with saliva sampling collection period specified as a random effect. Coefficient estimates, confidence intervals and *P*-value results for all models are shown in Fig. 3 and Table 2.

**Table 2:** Coefficient estimates, confidence interval (CI) and *P*-values values for variables in the cortisol, cortisol:DHEAS and DHEAS linear mixed models

	Coefficient estimate	CI (low)	CI (high)	*P*-value
**Cortisol model**				
Time of day	**0**.**19**	**0**.**17**	**0**.**22**	**<0**.**001**
Multiday seizure cycle	**1**.**19**	**1**.**02**	**1**.**39**	**0**.**029**
Pre-sample seizure	**1**.**32**	**1**.**03**	**1**.**71**	**0**.**030**
Post-sample seizure	0.93	0.72	1.21	0.598
PSS score	1.00	0.84	1.20	0.975
Allocated risk	0.91	0.79	1.06	0.204
**Cortisol:DHEAS model**				
Time of day	**0**.**19**	**0**.**16**	**0**.**22**	**<0**.**001**
Multiday seizure cycle	1.12	0.76	1.67	0.552
Pre-sample seizure	1.27	0.96	1.69	0.099
Post-sample seizure	0.88	0.66	1.18	0.400
PSS score	0.94	0.60	1.49	0.796
Allocated risk	0.97	0.67	1.41	0.670
**DHEAS model**				
Time of day	1.00	0.95	1.05	0.983
Multiday seizure cycle	1.04	0.71	1.52	0.822
Pre-sample seizure	1.05	0.95	1.16	0.348
Post-sample seizure	1.05	0.95	1.17	0.340
PSS score	1.07	0.66	1.71	0.784
Allocated risk	0.94	0.66	1.35	0.740

Cofficient estimates, confidence intervals and *P*-values are in bold font when significant (*P* < 0.05). Coefficient estimates and confidence intervals were exponentiated, giving a ratio measure of effect on the response variables.

For cortisol, the model explained 72% of the variance ($R_{\mathrm{conditional}}^{2}=0.72$) and 68% by fixed effects alone ($R_{\mathrm{marginal}}^{2}=0.68$). The effects of time of day, multiday seizure cycle and pre-sample seizure were statistically significant (*P* < 0.001, *P* = 0.029 and 0.030, respectively), with time of day explaining the majority of the variance (63%) and multiday seizure cycle and pre-sample seizure explaining an additional 1% each. Nevertheless, being at a seizure cycle peak was associated with a 19% increase in cortisol relative to a seizure cycle trough (OR = 1.19, 95% CI: 1.02–1.39). This effect remained significant under permutation testing, with the observed multiday seizure cycle coefficient exceeding approximately 97% of coefficients (*n* = 2000) obtained under shuffled and randomly assigned trough/peak labels (*P* = 0.022 and *P* = 0.038, respectively). The effect of time of day and multiday seizure cycle were also observed at the cohort level (Supplementary Figs 1 and 2). DHEAS and cortisol:DHEAS ratio models are described in the supplement. All results in the linear models were validated and reproduced using a Bayesian approach (Supplementary Table 3).

Three variations of the original cortisol model were derived to investigate other variables of interest (Supplementary Fig. 5). Consistent with the original model, all three variations revealed significant associations of time of day and multiday seizure cycle, with cortisol concentration. Pre-sample seizure (using 12 h window) was positively associated with cortisol concentration in all models except the model that incorporated time since waking. While this feature was not included in the main analysis due to missing observations (i.e. all participants wore a smartwatch, but adherence was ∼75%), it highlights the importance of tracking sleep timing when validating this work in future.

### Multiday seizure cycle and cortisol

**Figure 4:**
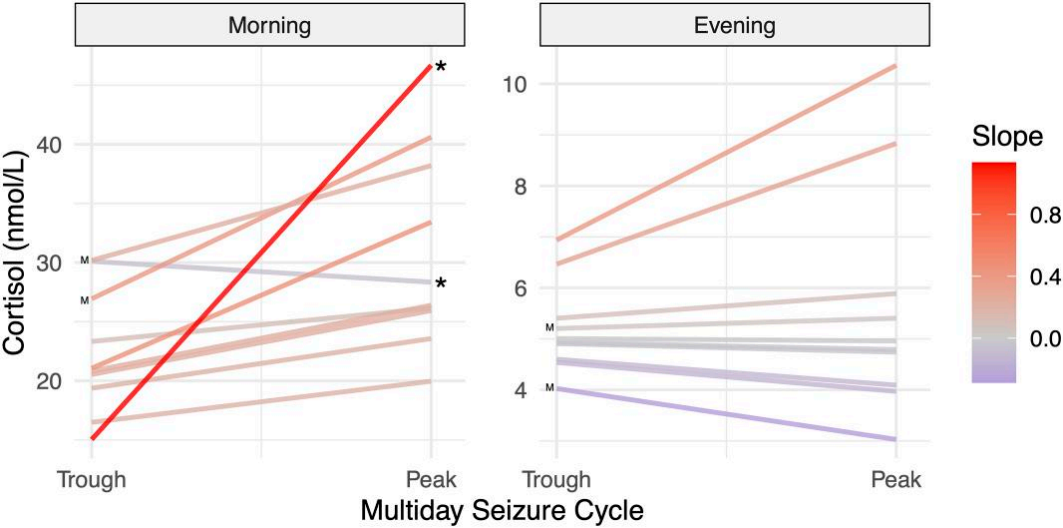
**Individual participant trajectories of morning (left) and evening (right) cortisol concentrations (nmol/L) at multiday cycle peaks and troughs.** Lines show fitted trajectories from individual-specific slopes estimated from the mixed effects model in Eq. 3, where the colour of the fitted line represents the strength of the slope. Each line is fitted to *n* = 10 observations. Significance (*P < 0.05) of these lines was tested using linear regression (*t*-test) analyses. *T*-statistic (*P*-value) for each significant slope: morning cortisol P9 = 6.38 (*P* < 0.001); morning cortisol P11 = −2.65 (*P* = 0.024). ‘M’ marks male participants.

Given the strong effect of time of day observed in the cortisol model, cortisol concentrations at multiday cycle peaks and troughs were analysed separately for morning and evening samples (Fig. 4). Morning cortisol levels were 37% higher (OR = 1.37, CI: 1.02–1.87) during multiday seizure cycle peaks compared with troughs, although this effect did not reach statistical significance (*P* = 0.07). When participants with weaker multiday seizure cycles (SI < 0.2, *n* = 2) were removed from the cohort, the effect became significant (OR = 1.53, 95% CI: 1.13–2.18, *P* = 0.04, *n* participants = 8), suggesting a possible ‘dose-dependent’ relationship whereby more distinct multiday rhythms in seizure timing correspond to stronger associations between cycle phase and morning cortisol. On an individual level, morning cortisol levels were elevated (i.e. positive slope) at multiday cycle peaks compared to troughs in 9 out of 10 participants (and all 8 participants with stronger cycles, Supplementary Fig. 6), although the trend was only significant in one participant. In evening saliva samples, there was no observed association between evening cortisol and cycle phase on a group or individual level; although the slope of this relationship was significantly influenced by intra-individual variance in cortisol (*P* = 0.013) (Supplementary Fig. 7 and Table 4). Means and standard deviations of cortisol samples across all sampling rounds, stratified by time of day and multiday cycle phase is shown in Supplementary Table 5.

**Figure 5:**
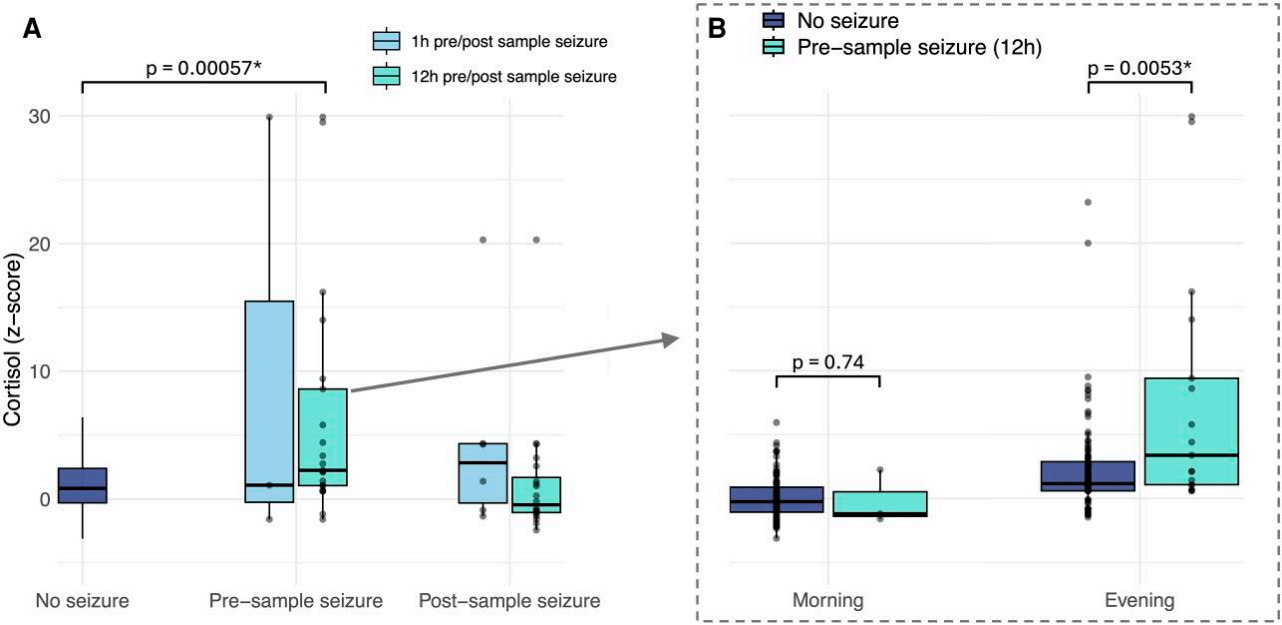
**(A) Standardized cortisol concentrations if no seizure occurred pre/post sample compared to samples where a seizure occurred before (pre-sample seizure) or after (post-sample seizure) the sample, and (B) standardized cortisol concentrations in the seizure versus 12 h pre-sample seizure condition, stratified by time of day.** (**A**) The baseline (‘No seizure’ condition; shown in dark blue, *n* = 203 total observations, observed in 10 participants) was compared to pre/post sample seizures using a 1-h (blue, *n* = 3 and *n* = 6 observations for pre/post, observed in three and four participants, respectively) and 12-h (green, *n* = 20 and *n* = 20 observations for pre/post, with both conditions observed in nine participants). (**B**) The baseline (‘No seizure’) condition was stratified by time of day of the samples (*n* = 102 morning and *n* = 101 evening, both conditions observed in 10 participants) and compared to the morning (*n* = 3, observed in three participants) and evening (*n* = 17, observed in eight participants) pre-seizure samples, respectively. Cortisol levels were standardized to the normal expected range of cortisol at 8 a.m. and 8 p.m. for morning and evening samples, respectively (Nutripath Pty Ltd). *P*-values were calculated using the Mann–Whitney–Wilcoxon test with Bonferroni correction applied across six comparisons (corrected significance threshold: *P* < 0.0083). Significant *P*-values are reported above the box plots. *W*-statistic (*P*-value) for each significant finding: no seizure versus pre-sample seizure (12 h) = 1081 (*P* < 0.001); no seizure versus pre-sample seizure (12 h) in evening samples = 496 (*P* = 0.0053).

To assess how much of this multiday cycle phase/cortisol relationship was simply driven by seizure timing (i.e. pre- and post-sample seizure), we used a mixed-effects logistic regression with random intercepts for participants to investigate the correlation between multiday cycle phase and seizure timing. The model revealed that both pre- and post-sample seizure occurrence (using 1-h and 12-h windows) was not significantly associated with being at a multiday seizure cycle peak; however, the trend was positive, which suggests that a larger cohort may be required to observe this effect. Nevertheless, seizure occurrence prior to saliva samples did appear to elevate cortisol levels on a cohort level (Fig. 5) when the pre-sample observation window was 12 h (*P* = 0.00057), which could also reflect the effect of the multiday cycle peak. However, when stratified by time of day, the effect remained significant only in the evening samples (*P* = 0.0053) and was not observed in morning samples (*P* = 0.74), suggesting that evening samples were affected to a greater extent by recent seizure occurrence. Note that these analyses are exploratory, given the small and unequal group sizes, and findings should be interpreted with caution pending replication in larger cohorts.

### Other analyses

Seizure frequency (in seizures per month, including seizures reported between participants’ first and final saliva collections) was also compared to stress hormone concentrations. No significant associations (*P* > 0.05) were found between seizure frequency and the mean or variance of participants’ stress hormone (DHEAS, morning cortisol and evening cortisol) concentrations throughout the study. However, the sparse nature of the hormone sampling limits the statistical power to compare the association between seizure burden and characteristic stress hormonal state.

The average PSS score across the epilepsy cohort was 19 (SD = 7, Males: 14.1 ± 6.6, Females: 21.3 ± 6.1) (Supplementary Fig. 8), which is higher than the general population (12.1 for males and 13.7 for females).^49^ Total PSS scores were significantly higher in females compared to males (Supplementary Fig. 9, *P* < 0.0001), consistent with previous findings.^41,49^ No significant relationship was observed between the peaks and troughs of multiday seizure cycles and total PSS scores (Supplementary Fig. 9), indicating that perceived stress does not vary systematically with multiday seizure cycles. Additionally, a sub-analysis of items on PSS questionnaire items and stress hormone concentrations revealed that item 9 (‘In the last month, how often have you been angered because of things that were outside of your control?’) was the only item to be positively correlated with DHEAS (*R* value = 0.304, *P* = 0.028) and cortisol levels (*R* value = 0.124, *P* = 0.028) (Supplementary Fig. 10).

## Discussion

This study investigated stress hormones and multiday seizure cycles in a longitudinal pilot study of ten individuals with epilepsy. While the sample size is modest and multiday seizure cycles varied in strength across participants, these data provide a pivotal step towards characterizing the dynamic interplay between cortisol and seizure susceptibility over extended timescales. Disentangling the contributions of circadian and multiday rhythms, in addition to seizure occurrence, to cortisol variation is inherently challenging and necessitated the range of statistical tests presented in this work. While the circadian cycle is the primary driver of cortisol, the present results suggest that cortisol may also exhibit multiday fluctuations in people with epilepsy, potentially shedding light on the mechanisms underpinning multiday rhythms in epilepsy.

Across the cohort, cortisol levels were significantly higher during multiday cycle peaks (increased seizure susceptibility) compared to cycle troughs (decreased susceptibility), particularly in morning samples, an effect that appeared to be more pronounced in individuals with stronger cyclical trends in seizure timing. This increased cortisol may be partly induced by seizure occurrence, as the current work found significant post-ictal (up to 12 h) changes in evening cortisol concentration; a finding that is supported by previous studies.^7,11^ However, the current study found that only evening salivary cortisol levels were significantly elevated after seizures, nor was seizure frequency correlated with overall cortisol. Therefore, it is possible that, at least in some individuals with epilepsy, the increase in morning cortisol observed at cycle peaks is associated with cyclic patterns of seizure susceptibility,^7,11^ irrespective of seizure occurrence. Future studies incorporating more reliable multiday cycle estimates (e.g. interictal spike recordings) alongside repeated stress hormone measurements will be essential to more fully characterize this relationship and reduce uncertainty in cycle phase assignment.

Previous studies that investigated stress hormones in epilepsy primarily assessed either baseline cortisol and/or DHEAS levels in people with epilepsy, compared to healthy controls, or investigated short-term changes in cortisol before and after seizures.^7^ Most studies show an acute, post-ictal increase in cortisol, suggesting seizures act as a stressor, although this effect has primarily been demonstrated from serum cortisol. Notably, a study by van Campen *et al*.^33^ assessed the temporal correlation between salivary (circulating) cortisol and rate of epileptiform discharges, revealing their relationship over circadian timescales, particularly in people who reported stress-sensitive seizures. The current study expands this work by considering the multiday dynamics between cortisol and seizures.

Very few previous studies have assessed long-term, individual changes in cortisol. One early study assessed longitudinal serum cortisol at 3-month intervals in 45 patients with newly diagnosed epilepsy and found pronounced variability in group average cortisol levels over the 2-year study, despite consistent anti-seizure medication dose.^15^ Unfortunately, the aforementioned study did not report on individual trends, and group-level variability may have been driven by intra-individual multiday fluctuations in cortisol. To our knowledge, no other studies have tracked cortisol dynamics in people with epilepsy over multiple days. However, a longitudinal study tracked daily self-reported stress for over a year in 71 people with epilepsy^6^ and found stress levels were positively associated with seizure risk. Other studies of physiological signals linked to the stress response (heart rate, electrodermal activation, skin temperature) have also identified intrinsic multiday rhythms linked to seizure cycles.^29,30^ These previous studies, alongside the presented results, highlight the importance of considering dynamic interactions between stress and seizures.

In addition to disruption of cortisol associated with seizure occurrence or seizure cycles, people with epilepsy may exhibit higher baseline levels of stress hormones compared to control groups.^12-14^ Furthermore, people with epilepsy show increased self-reported stress and anxiety compared to the general population.^50^ The current cohort showed higher cortisol levels compared to the distribution of the expected general population, regardless of when cortisol was measured (i.e. at the peak or trough of their seizure cycle). These findings support previous studies proposing epilepsy as a model for chronic stress, with further sensitization induced by seizures.^7-11^ Perceived stress was also higher in the epilepsy cohort than the general population, and higher in females than males, consistent with previous reports.^41^ Interestingly perceived stress was not linked to seizure cycles or stress hormones (cortisol or DHEAS), suggesting that the observed changes in stress hormones were not detectable by individuals. Indeed, if multiday hormonal fluctuations do exist, potentially reflecting an intrinsic homeostatic process, then it would be counterproductive for these oscillations to induce stress. Emerging technologies that continuously sample hormone levels may offer promise for future studies to investigate whether cortisol does indeed fluctuate in multiday patterns.^51^

Hormonal dynamics are inherently oscillatory, and these dynamics are essential to healthy function.^52^ The ultradian (pulsatile) and circadian rhythmicity of cortisol is well studied, and longer oscillations have been observed in timing and amplitude of cortisol secretion, including seasonal, menstrual and weekday changes.^53,54^ While the purpose of longer, periodic fluctuations in hormone levels remains unclear, it has been hypothesized that they provide an adaptive advantage to respond to changing environmental demands, and similar rhythms have been observed in other biomarkers of healthy individuals, including blood pressure, basal temperature and immune activation.^55^ While the current study was not powered to definitively establish intrinsic multiday rhythmicity of cortisol, the observed patterns suggest that multiday fluctuations in cortisol are preserved in epilepsy. In this cohort, overall cortisol levels were elevated and morning cortisol appeared to covary with seizure cycles, with peak seizure risk times tending to correspond to increased morning cortisol. Importantly, individuals with stronger seizure cycles appeared to have a stronger association between seizure cycle phase and cortisol concentrations, suggesting that screening for participants with stronger multiday cycles in future may enhance the findings. Conversely, evening cortisol was not correlated with multiday seizure cycles but, unlike morning cortisol, it was correlated to seizure occurrence. This difference in morning versus evening samples may reflect a potential tendency for evening cortisol to be influenced by seizures occurring around the time of sampling, as well as other acute stressors encountered during the day, compared to morning cortisol.

It is possible that seizure susceptibility is increased at times when cortisol is naturally elevated or, conversely, other factors that heighten seizure risk may also modulate cortisol. In the latter case, disruption of cortisol dynamics may cause further damage, as networks underlying hormonal secretion are highly sensitive to the timing of stressors.^52^ For instance, perturbations of the hypothalamic-pituitary-adrenal axis and its coupled oscillators may induce longer-term disruptions to sleep, menstruation and fertility.^52,56^ More generally, misalignment of hormonal rhythms is implicated in a range of diseases along with overall morbidity/mortality. In addition, perfectly decoupling the influence of the seizure cycle on hormonal levels from the influence of seizure events is challenging in patients with drug-resistant epilepsy. While further work is needed to directly probe whether multiday fluctuations in cortisol drive or are co-modulated with seizure cycles, it may be necessary to investigate this in individuals with drug-responsive epilepsy who are seizure-free or in individuals with less frequent seizures.

A major limitation of this preliminary study was the reliance on self-reported seizure timing to establish seizure cycles. Although cycles of seizure diaries can reliably correlate with cycles estimated from electrographic seizures,^28^ it is likely that both over- and under-reporting, particularly of nocturnal seizures, affected the results. For instance, a more accurate record of seizure times and tracking an underlying continuous seizure risk biomarker (e.g. interictal spikes) would substantially improve the model’s ability to detect seizure-related changes in cortisol. On the other hand, the use of self-reported seizures allows individuals with stronger cycles to be identified from routinely collected clinical data, potentially allowing targeted interventions aimed at mitigating stress for patients who may be most likely to benefit. Furthermore, previous studies have identified acute post-ictal cortisol increases following generalized tonic-clonic and focal impaired awareness seizures.^46^ Future studies with sufficient power to distinguish between these seizure types will be important for refining interpretation of the present findings. There was, however, a significant trend for increased evening cortisol within 12 h of seizures, which may become more pronounced with more accurate seizure capture. Similarly, allocated high-/low-risk periods were not associated with changes in cortisol, suggesting that prospective assignment of risk based on seizure diaries was not sensitive enough to track epilepsy nor autonomic imbalance. Nevertheless, while this work is not intended to be a validation of the cycle forecasting model, it is important to note that prospectively scheduling during high-risk states led to a 3.1-fold higher chance of being at a retrospectively-mapped multiday seizure cycle peak. This suggests that prospective scheduling is significantly more powerful than random chance at predicting future cycle peaks and troughs.

Further work is underway to replicate the current study design in cohorts with chronic, implanted EEG recordings to assess the relationship between multiday cycles of interictal discharges, seizures and stress. Furthermore, this study did not consider the effect of anti-seizure medications on cortisol, and individuals were on diverse treatments including monotherapy (four individuals), polytherapy (seven individuals) or no medications (two individuals). Previous studies have shown anti-seizure medications may affect hormone levels, as well as seizure cycles,^28,57^ so further work is necessary to investigate these effects on the stress-seizure relationship. Similarly, the cohort was too small to robustly determine whether other demographic or clinical factors impact the findings relating stress and seizure cycles.

## Conclusion

The current study revealed important preliminary insights into the stress-seizure dynamic over slow timescales, providing the first line of evidence that stress hormones may co-oscillate with multiday rhythms of seizure susceptibility, irrespective of perceived stress levels. This dissociation between perceived stress and physiological stress responses suggests multiday hormonal fluctuations reflect an intrinsic homeostatic process, which may be disrupted by external stressors like seizures. The results underscore the need for further longitudinal studies to shed light on the drivers of multiday physiological cycles in epilepsy, paving the way for more targeted interventions during periods of increased seizure susceptibility.

## Supplementary Material

fcag217_Supplementary_Data

## Data Availability

Requests for data can be made to the corresponding author. Deidentified data will be made available upon reasonable request. Code used for analysis (statistics and figure generation) is available at: https://github.com/RiPLresearch/cortisol-study-brain-communications.
