# Supplementary material for: Investigation of stress hormones across multiday seizure cycles
Source: Brain Commun. 2026 Jun 8;8(3):fcag217. doi: 10.1093/braincomms/fcag217 (PMC13270484; doi:10.1093/braincomms/fcag217)
Supplement: fcag217_Supplementary_Data [file fcag217_supplementary_data.pdf]

Supplementary Materials

Supplementary Table 1. 10-item Perceived Stress Scale scoring system. Total score is the sum of scores for all responses.

**Negative items (1, 2, 3, 6, 9, 10)**		**Positive items (4, 5, 7, 8)**	
**Possible response**	**Score per response**	**Possible response**	**Score per response**
Never	0	Never	4
Almost never	1	Almost never	3
Sometimes	2	Sometimes	2
Fairly often	3	Fairly often	1
Very often	4	Very often	0

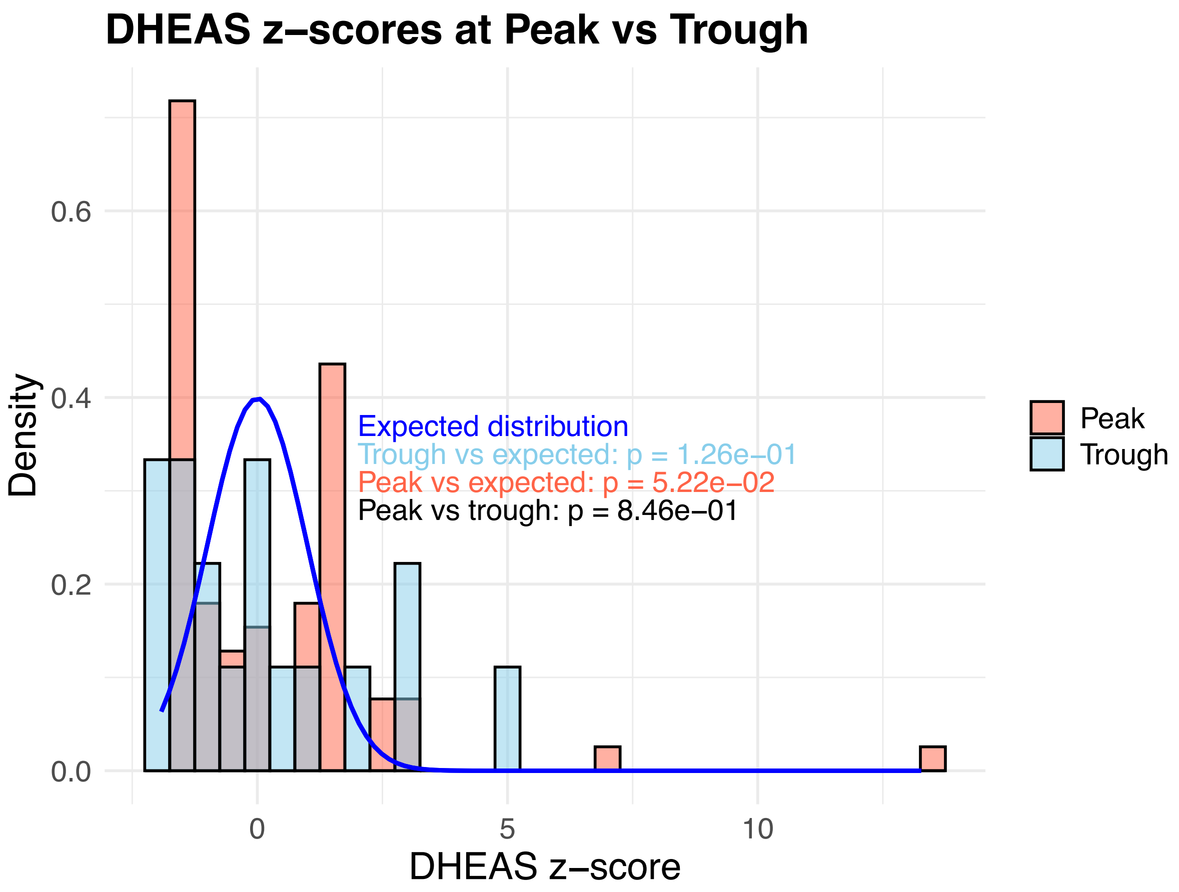

Supplementary Figure 1. Dehydroepiandrosterone Sulphate (DHEAS) levels in the epilepsy population at multiday seizure cycle peak vs trough, compared to the expected general population. The blue histogram of represents all participants' z-scored DHEAS concentrations collected during the trough of their multiday cycle (n=17 observations), whereas the red histogram represents saliva samples collected during the peak of their multiday cycle (n=23 observations). The dark blue "Expected distribution" line represents expected distribution of DHEAS levels across the general population (Nutripath Pty Ltd). Differences in the distributions were tested using the Kolmogorov-Smirnov test and p-values (of which none were significant). Note that DHEAS concentrations were only measured in the first saliva sample of each sampling collection period (i.e. Day 1, 8 AM), thus only 4 DHEAS saliva collections were obtained per person.

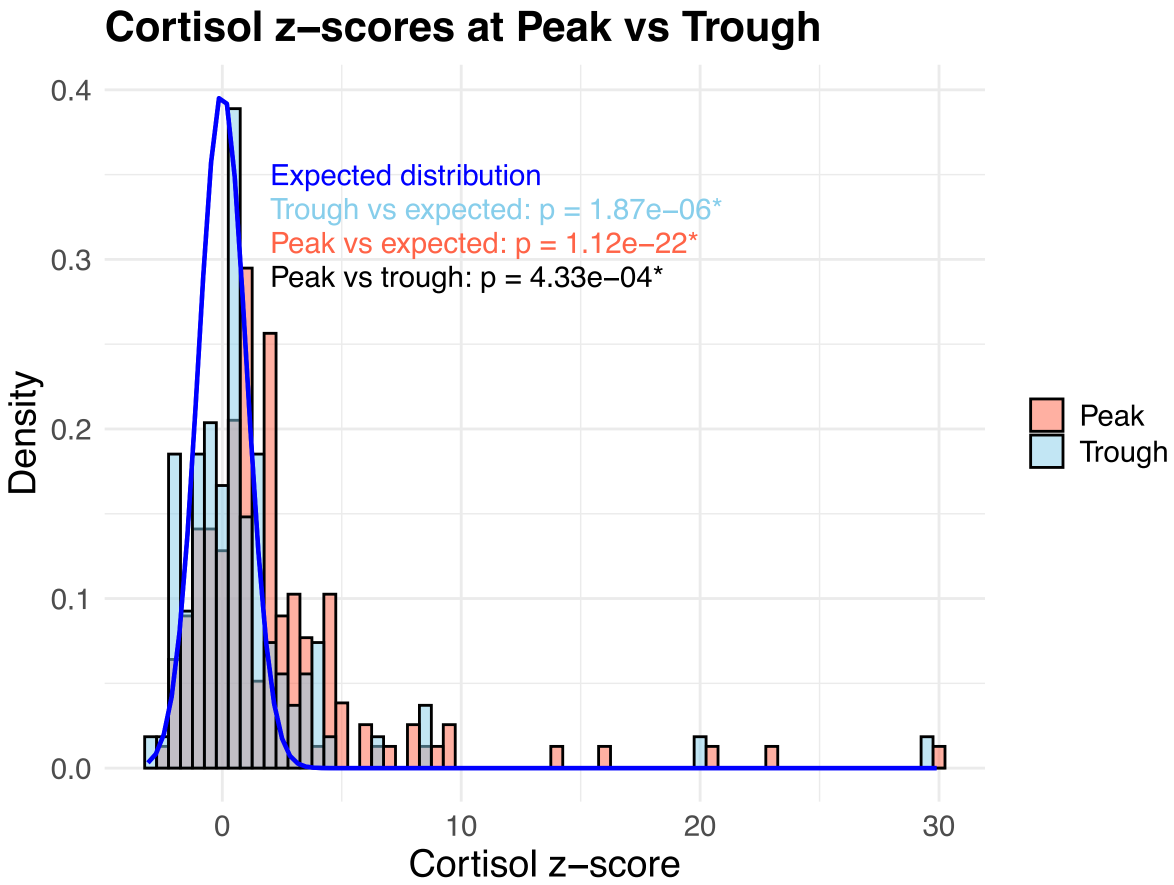

Supplementary Figure 2. Cortisol levels in the epilepsy population at multiday seizure cycle peak vs. trough, compared to the expected general population. The blue histogram of represents all participants' z-scored cortisol concentrations taken from saliva samples collected during the trough of their multiday cycle (n=102 observations), whereas the red histogram represents saliva samples collected during the peak of their multiday cycle (n=138 observations). The dark blue "Expected distribution" line represents expected distribution of cortisol levels across the general population (provided by Nutripath Pty Ltd). Differences in the distributions were tested using the Kolmogorov-Smirnov test and p-values (* indicating significance at $p < 0.05$) are reported. D-statistic [p-value] for each significant finding: Trough vs expected cortisol distribution = 0.25 [$p < 0.001$]; Peak vs expected cortisol distribution = 0.41 [$p < 0.001$]; Peak vs Trough = 0.26 [$p < 0.001$].

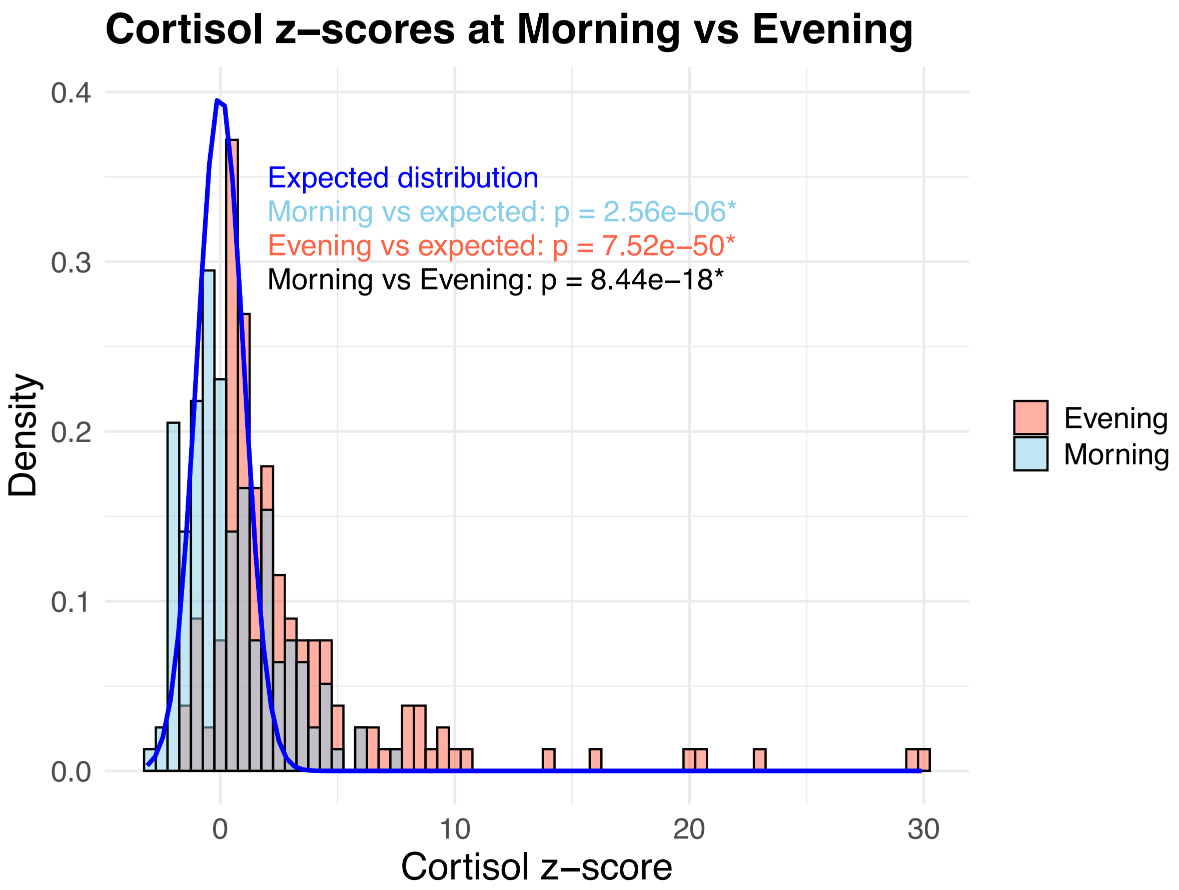

Supplementary Figure 3. Cortisol levels in the epilepsy population in morning vs. evening samples, compared to the expected general population. The blue histogram of represents all participants' z-scored cortisol concentrations (standardized to the normal expected range of cortisol at 8AM) taken from saliva samples collected during the morning (n=156 observations), whereas the red histogram represents saliva samples collected during the evening (n=156 observations, standardized to the normal expected range of cortisol at 8PM). The dark blue "Expected distribution" line represents expected distribution of cortisol levels across the general population (Nutripath Pty Ltd). Differences in the distributions were tested using the Kolmogorov-Smirnov test and p-values (* indicating significance at $p < 0.05$) are reported. D-statistic [p-value] for each significant finding: Morning sample vs expected cortisol distribution = 0.21 [$p < 0.001$]; Evening sample vs expected cortisol distribution = 0.60 [$p < 0.001$]; Morning vs Evening = 0.50 [$p < 0.001$].

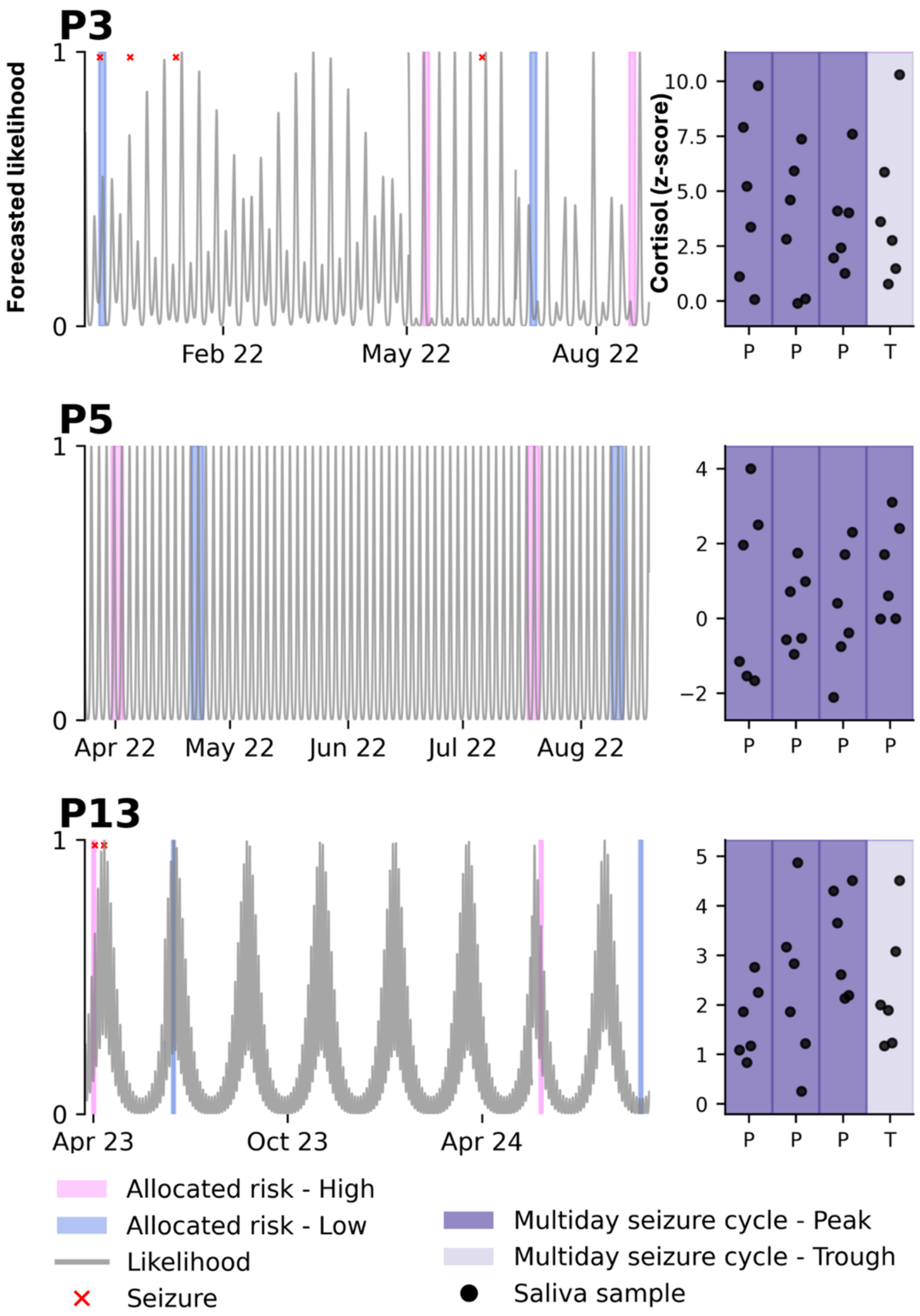

Supplementary Figure 4. Prospective cycle forecast and allocated risk (left panels), and corresponding multiday seizure cycle phase with standardized cortisol levels (right panels). Only participants excluded from the retrospective analyses are shown (n=3, participants P3, P5 and P13). For each participant, the left panel shows the prospective likelihood from the cycle forecast (grey line), with reported seizures overlaid at the top border (red crosses). Shaded regions indicate the four scheduled sampling periods, coloured by allocated risk (pink: high risk; blue: low risk). The right panel displays standardized cortisol concentrations (z-scores; black markers) measured during the scheduled sampling periods (n=24 per participant). Background shading in the right panel represents the retrospective multiday seizure cycle phase during each sampling window (dark purple: cycle peak (P); light purple: cycle trough (T)). In both panels, shaded regions correspond to the same sampling periods, enabling direct visual comparison between allocated risk, multiday seizure cycle and observed cortisol dynamics.

Supplementary Table 2. Cycle and forecast characteristics of study participants. Cycle period is reported in days, and cycle strength is the Synchronization Index (SI).

Participant	Multiday seizure cycle period	Multiday seizure cycle strength	Allocated risk cycle periods and strengths
P1	7	0.14	6.0 days (SI=0.20), 44.0 days (SI=0.32)<br>4.25 days (SI=0.19), 19.5 days (SI=0.19)<br>7.0 days (SI=0.19), 15.0 days (SI=0.19)<br>7.0 days (SI=0.18), 8.0 days (SI=0.22)
P2	10.75	0.36	3.5 days (SI=0.46), 56.0 days (SI=0.54)<br>2.5 days (SI=0.45), 52.5 days (SI=0.57)<br>2.5 days (SI=0.40), 51.0 days (SI=0.55)<br>2.5 days (SI=0.43), 53.0 days (SI=0.59)
P4	13.75	0.36	6.75 days (SI=0.39), 15.0 days (SI=0.43)<br>6.75 days (SI=0.40), 15.5 days (SI=0.39)<br>6.75 days (SI=0.33), 21.0 days (SI=0.36)<br>5.25 days (SI=0.30), 21.0 days (SI=0.39)
P6	7	0.39	7.0 days (SI=0.53), 45.0 days (SI=0.38)<br>7.0 days (SI=0.36), 45.5 days (SI=0.39)<br>7.0 days (SI=0.38), 25.0 days (SI=0.42)<br>6.0 days (SI=0.31), 25.5 days (SI=0.40)
P7	22	0.34	4.25 days (SI=0.22), 22.0 days (SI=0.36)<br>4.25 days (SI=0.22), 22.0 days (SI=0.36)<br>6.5 days (SI=0.24), 34.5 days (SI=0.44)<br>6.25 days (SI=0.23), 33.5 days (SI=0.42)
P8	7	0.24	7.0 days (SI=0.25), 25.5 days (SI=0.11)<br>7.0 days (SI=0.27), 66.5 days (SI=0.14)<br>7.0 days (SI=0.23), 66.5 days (SI=0.14)<br>3.5 days (SI=0.20), 25.5 days (SI=0.12)'
P9	4.75	0.21	4.75 days (SI=0.20), 12.0 days (SI=0.23)<br>4.75 days (SI=0.24), 12.0 days (SI=0.22)<br>4.75 days (SI=0.26), 12.0 days (SI=0.15)<br>4.75 days (SI=0.28), 13.5 days (SI=0.17)
P10	13.75	0.82	2.0 days (SI=0.62), 12.5 days (SI=0.78)<br>5.75 days (SI=0.50), 34.5 days (SI=0.69)<br>6.75 days (SI=0.47), 12.5 days (SI=0.68)<br>6.75 days (SI=0.47), 12.5 days (SI=0.68)
P11	5.25	0.15	3.5 days (SI=0.27), 15.0 days (SI=0.21)<br>5.25 days (SI=0.27), 20.0 days (SI=0.24)<br>6.75 days (SI=0.33), 8.0 days (SI=0.36)<br>5.25 days (SI=0.39), 8.0 days (SI=0.39)
P12	13	0.46	4.75 days (SI=0.69), 13.0 days (SI=0.64)<br>7.0 days (SI=0.44), 13.0 days (SI=0.57)<br>3.25 days (SI=0.46), 21.5 days (SI=0.57)<br>5.5 days (SI=0.53), 13.0 days (SI=0.58)

Linear mixed models

Linear mixed models (Equation 2) were fitted to predict log-transformed Cortisol concentration, Cortisol:DHEAS ratio and DHEAS concentration using the fixed effects of time of day, multiday seizure cycle, allocated risk, pre-sample seizure, post-sample-seizure and PSS score, with saliva sampling collection period specified as a random effect. Coefficient estimates, confidence intervals and p-value results for all models are shown in Figure 2 and Table 2. For DHEAS, the model's explanatory power was substantial ($R^2_{\text{conditional}} = 0.96$), but the contribution from fixed effects was negligible ($R^2_{\text{marginal}} = 0.00654$) and none of the variables had a statistically significant effect ($p > 0.05$). For Cortisol:DHEAS ratio, the model explained 80% of the variance ($R^2_{\text{conditional}} = 0.80$) and 46% by fixed effects alone ($R^2_{\text{marginal}} = 0.46$). Like Cortisol, the effect of time of day was significant ($p < 0.001$), which may be entirely explained by the proportional relationship between Cortisol and Cortisol:DHEAS ratio. All results in the linear models were validated and reproduced using a Bayesian approach (Supplementary Table 3).

Supplementary Table 3. Bayesian analysis. Bayesian coefficient estimates and confidence intervals (CI) for variables in the Cortisol, Cortisol:DHEAS and DHEAS models. Significant ($p < 0.05$) values are in bold font. Coefficient estimates and confidence intervals were exponentiated, giving a ratio measure of effect on the response variables.

		**Coefficient estimate**	**CI (low)**	**CI (high)**
**Cortisol model**	Time Of Day	**0.19**	**0.17**	**0.22**
	Multiday Seizure Cycle	**1.19**	**1.01**	**1.41**
	Pre-Sample Seizure	**1.32**	**1.03**	**1.69**
	Post-Sample Seizure	0.94	0.72	1.21
	PSS Score	1.00	0.83	1.21
	Allocated Risk	0.91	0.78	1.06
**Cortisol:DHEAS model**	Time Of Day	**0.19**	**0.16**	**0.22**
	Multiday Seizure Cycle	1.11	0.75	1.65
	Pre-Sample Seizure	1.27	0.96	1.68
	Post-Sample Seizure	0.89	0.66	1.18
	PSS Score	0.94	0.60	1.48
	Allocated Risk	0.97	0.66	1.43
**DHEAS model**	Time Of Day	1.00	0.95	1.05
	Multiday Seizure Cycle	1.04	0.71	1.54
	Pre-Sample Seizure	1.05	0.95	1.16
	Post-Sample Seizure	1.05	0.95	1.16
	PSS Score	1.06	0.66	1.69
	Allocated Risk	0.94	0.65	1.35

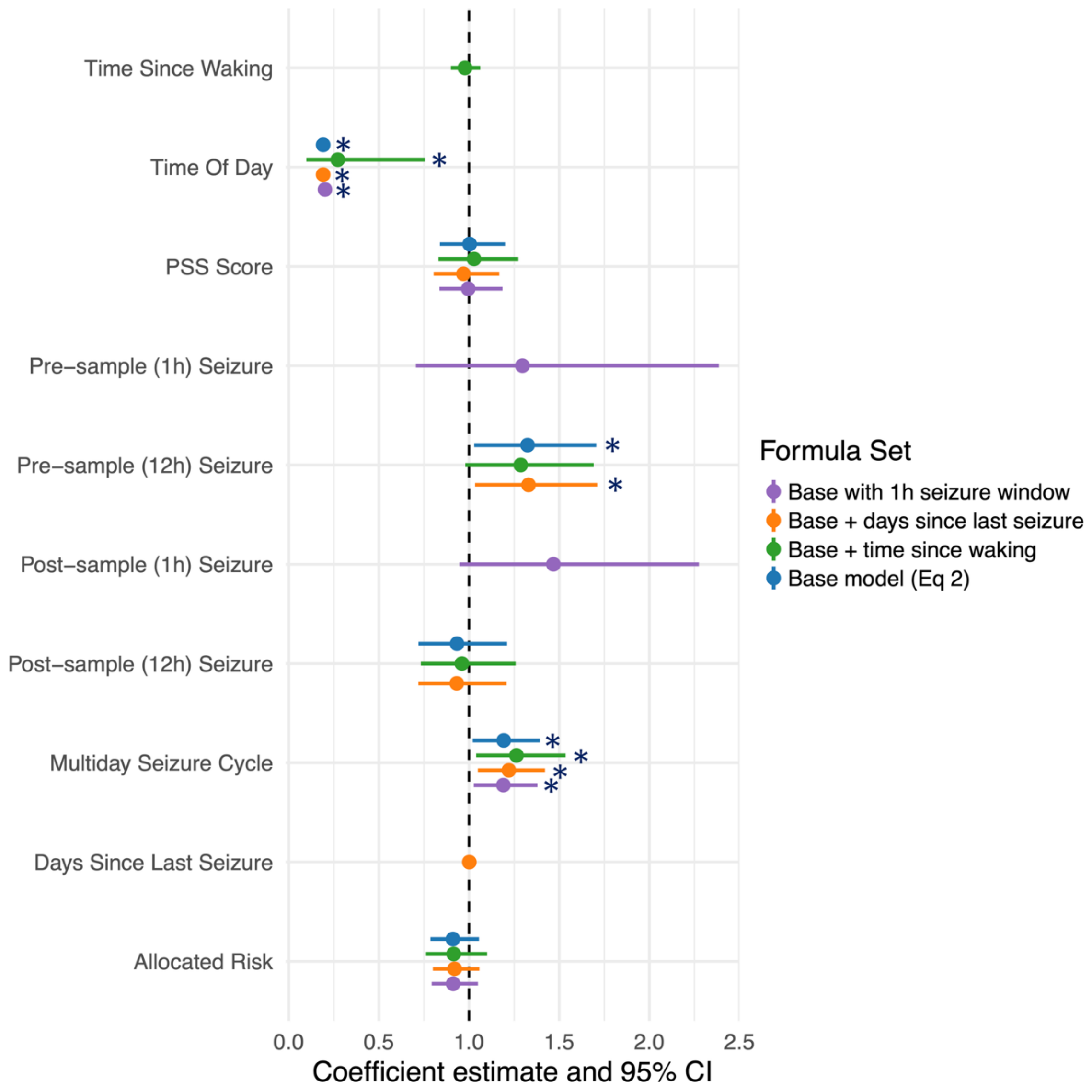

Supplementary Figure 5. Coefficient estimates and confidence intervals for fixed effects in four different linear models predicting cortisol concentration. The four models were: (1) the base model (blue), given in Equation 2 (Eq 2) of the manuscript (n=240 observations); (2) the base model with an additional independent variable "Time Since Waking" (green) (n=177 observations); (3) the base model with additional independent variable "Days Since Last Seizure" (orange) (n=240 observations); and (4) the base model with the pre- and post-sample seizure window increased to 12 hours (purple) (n=240 observations). Coefficient estimates and 95% confidence intervals were exponentiated, giving a ratio measure of effect on the response variables. *Indicates significance of the coefficient ($p < 0.05$), derived from Wald t-tests. Wald t-statistic [p-value] for each significant coefficient: Base model (Eq 2)/Time of Day = −23.36 [$p < 0.001$]; Base model (Eq 2)/Multiday Seizure Cycle = 2.30 [$p = 0.029$]; Base model (Eq 2)//Pre-sample (12h) Seizure = 2.19 [$p = 0.030$]; Base + time since waking/Time of Day = −2.53 [$p = 0.013$]; Base + time since waking/Multiday Seizure Cycle = 2.49 [$p = 0.021$]; Base + days since last seizure/Time of Day = −23.42 [$p < 0.001$]; Base + days since last seizure /Multiday Seizure Cycle = 2.59 [$p = 0.010$]; Base + days since last seizure/Pre-sample (12h) Seizure = 2.23 [$p = 0.027$]; Base with 1h seizure window/Time of Day = −23.57 [$p < 0.001$]; Base with 1h seizure window/Multiday Seizure Cycle = 2.32 [$p = 0.021$].

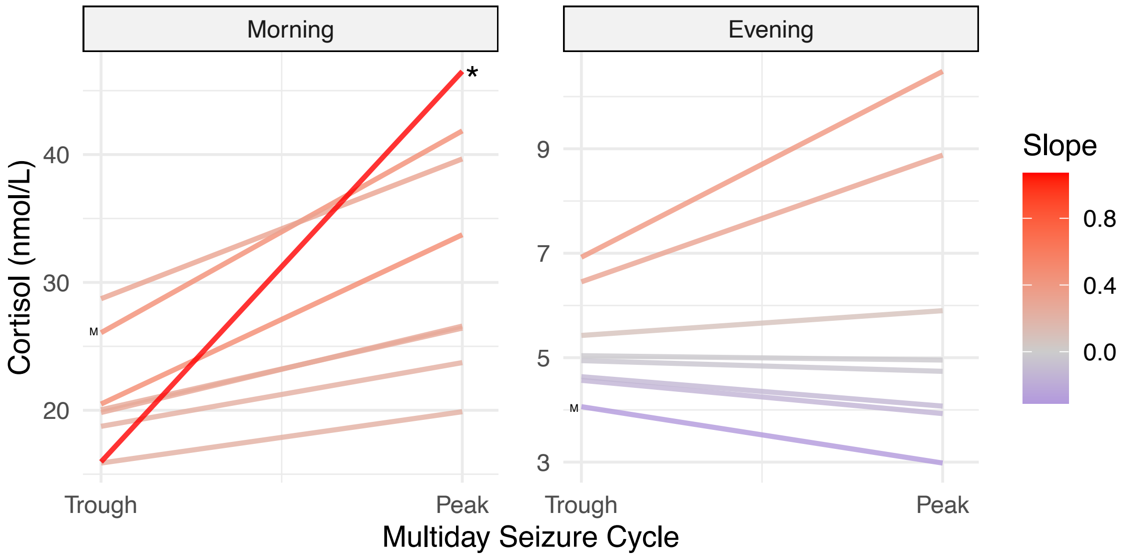

Supplementary Figure 6. Individual participant trajectories of morning (left) and evening (right) cortisol concentrations (nmol/L) at multiday cycle peaks and troughs for participants with stronger seizure multiday cycles (Synchronisation Index, SI ≥ 0.2). Lines show fitted trajectories from individual-specific slopes estimated from the mixed effects model in Equation 3, where the colour of the fitted line represents the strength of the slope. Each line is fitted to n=8 observations. Significance (*, $p < 0.05$) of these lines was tested using linear regression (t-test) analyses. T-statistic [p-value] for each significant slope: P9 = 6.38 [$p<0.001$]. "M" marks male participants.

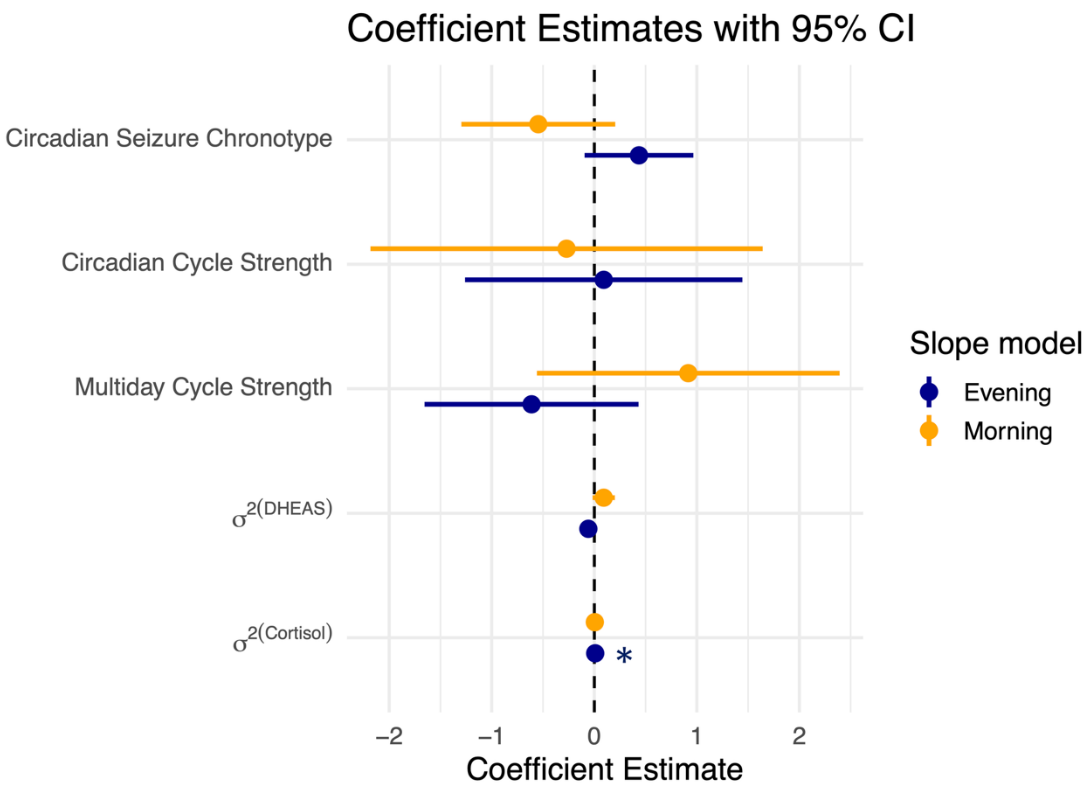

Supplementary Figure 7. Coefficient estimates and confidence intervals for fixed effects in the linear models predicting the slope of cortisol change across multiday seizure cycles (n=120 observations per slope model). Two models were evaluated and plotted together: morning slopes (orange) and evening slopes (blue). Variance symbols (σ^2) represent individuals' variances in Dehydroepiandrosterone Sulphate (DHEAS) and cortisol concentrations across all saliva samples. Circadian Cyle Strength and Multiday Cycle Strength represent the magnitude of the Synchronization Index for the circadian cycle (24 hour) and the strongest multiday (4 – 200 day) cycle, respectively. Circadian Seizure Chronotype represents the tendency of individuals' seizures to occur in the morning or afternoon, using the direction of the mean resultant vector (1=morning seizures, occurring between 12AM and 12PM; 0=afternoon seizures, occurring between 12PM and 12AM). Coefficient estimates, confidence intervals and p-values are reported in Supplementary Table 4. *Indicates significance of the coefficient ($p < 0.05$), derived from Wald t-tests. Wald t-statistic [p-value] for the significant coefficient: Evening/$\sigma^{2(Cortisol)}$= 4.28 [$p = 0.013$].

Supplementary Table 4. Coefficient estimates, confidence interval (CI) and p-values values for fixed effects in the linear models predicting the slope of cortisol change across multiday seizure cycles. Significant ($p < 0.05$) values are in bold font.

		Coefficient estimate	CI (low)	CI (high)	p-value
**Morning cortisol slope model**	σ^2(DHEAS)	-0.06	-0.14	0.02	0.103
	σ^2(Cortisol)	**0.01**	**0.00**	**0.01**	**0.013**
	Multiday Cycle Strength	-0.61	-1.66	0.43	0.179
	Circadian Cycle Strength	0.09	-1.26	1.44	0.860
	Circadian Seizure Chronotype	0.44	-0.09	0.97	0.085
**Evening cortisol slope model**	σ^2(DHEAS)	0.09	-0.02	0.20	0.080
	σ^2(Cortisol)	0.00	0.00	0.01	0.157
	Multiday Cycle Strength	0.92	-0.56	2.39	0.160
	Circadian Cycle Strength	-0.27	-2.18	1.64	0.714
	Circadian Seizure Chronotype	-0.55	-1.30	0.20	0.113

Supplementary Table 5. Mean and standard deviation of cortisol samples across all sampling rounds for participants included in the main analyses (n=10), stratified by morning and evening samples and saliva samples collected at retrospective multiday cycle troughs and peaks.

	**Morning**		**Evening**	
	**Trough**	**Peak**	**Trough**	**Peak**
**P1**	**24.4 ± 3.6**	**28 ± 15.4**	**4.1 ± 0.8**	**5.3 ± 1.5**
Round 1	23.2 ± 1.7		3.7 ± 0.1	
Round 2	25.6 ± 5		4.6 ± 1	
Round 3		22.4 ± 14.4		4.4 ± 1.2
Round 4		33.5 ± 17.2		6.2 ± 1.2
**P2**	**34.4 ± 20.7**	**47 ± 13.4**	**5.7 ± 3.8**	**2.7 ± 1**
Round 1		40.2 ± 16.7		3 ± 1.4
Round 2	31.2 ± 27.6		8.2 ± 4.1	
Round 3	37.7 ± 16.5		3.1 ± 0.8	
Round 4		53.7 ± 6		2.3 ± 0.6
**P4**	**21.6 ± 8.6**	**37.2 ± 15.7**	**9.7 ± 11.5**	**13.1 ± 6.9**
Round 1		38.8 ± 9.5		15.4 ± 9.4
Round 2		42.5 ± 22.5		8.7 ± 2.6
Round 3	21.6 ± 8.6		9.7 ± 11.5	
Round 4		30.1 ± 16.5		15.2 ± 7.2
**P6**	**24.1 ± 19.2**	**20.2 ± 9.7**	**6.2 ± 4**	**6.8 ± 3.8**
Round 1	24.1 ± 19.2		6.2 ± 4	
Round 2		21.2 ± 7.9		4.5 ± 1.2
Round 3		18.9 ± 13.8		6.6 ± 4
Round 4		20.6 ± 10.9		9.4 ± 4.8
**P7**	**21.8 ± 8**	**23.5 ± 3.1**	**4.4 ± 0.9**	**3.7 ± 0.2**
Round 1	22.4 ± 8.6		4.7 ± 1.4	
Round 2		23.5 ± 3.1		3.7 ± 0.2
Round 3	26.9 ± 4.5		4.4 ± 0.3	
Round 4	16 ± 8.6		4.2 ± 1	
**P8**	**35.3 ± 8.9**	**43.4 ± 14.3**	**4.4 ± 1.2**	**4.2 ± 1.5**
Round 1	27.7 ± 4.9		5.1 ± 1.4	
Round 2		45.2 ± 21.4		4 ± 2.2
Round 3	42.9 ± 1.9		3.6 ± 0	
Round 4		41.7 ± 6.6		4.4 ± 0.7
**P9**	**10.5 ± 2.3**	**56.7 ± 18.9**	**16.2 ± 14.6**	**11 ± 9.3**
Round 1		51.2 ± 10.6		10.3 ± 7.8
Round 2		45.5 ± 24.2		14.8 ± 15.7
Round 3	10.5 ± 2.3		16.2 ± 14.6	
Round 4		73.3 ± 8.8		7.9 ± 2.1
**P10**	**22 ± 13.3**	**22.8 ± 7.7**	**4.8 ± 1.6**	**5.3 ± 1.5**
Round 1		29.6 ± 2.4		6.3 ± 1.6
Round 2	10.5 ± 0.6		3.9 ± 0.5	
Round 3		16 ± 2.1		4.3 ± 0.7
Round 4	33.5 ± 6.8		5.7 ± 1.8	
**P11**	**35.2 ± 6.8**	**27.4 ± 3.4**	**6.2 ± 1.1**	**5.2 ± 1.3**
Round 1		26.2 ± 4.7		6.1 ± 1.3
Round 2	30.8 ± 2.8		6.7 ± 0.3	
Round 3		28.7 ± 1.2		4.3 ± 0.7
Round 4	39.6 ± 7.1		5.8 ± 1.6	
**P12**	**21 ± 6.3**	**27.4 ± 10.6**	**3.9 ± 0.4**	**5.6 ± 4.3**
Round 1		16.8 ± 1.1		8.3 ± 7.5
Round 2		34.6 ± 10.9		3.7 ± 0.1
Round 3	21 ± 6.3		3.9 ± 0.4	
Round 4		30.8 ± 8.2		4.7 ± 0.1

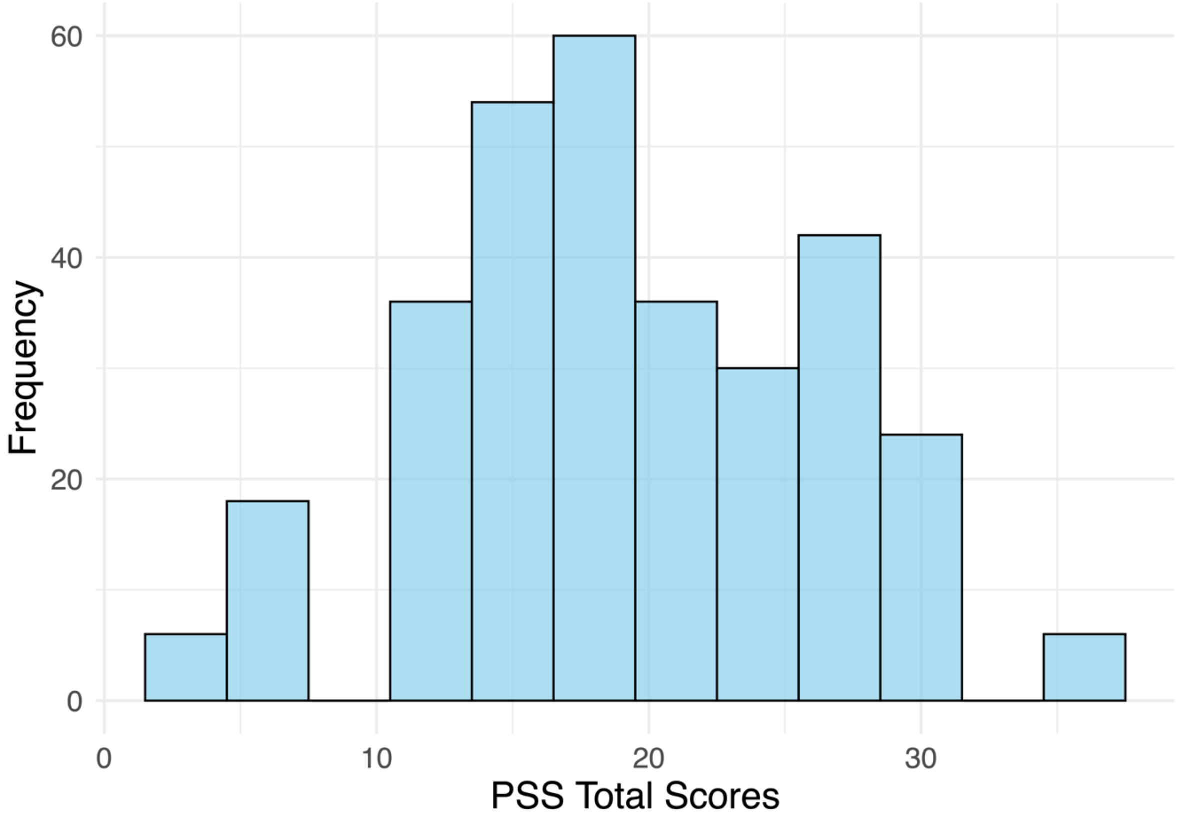

Supplementary Figure 8. Histogram of participants' Perceived Stress Scale (PSS) scores across all time periods (n=52 observations).

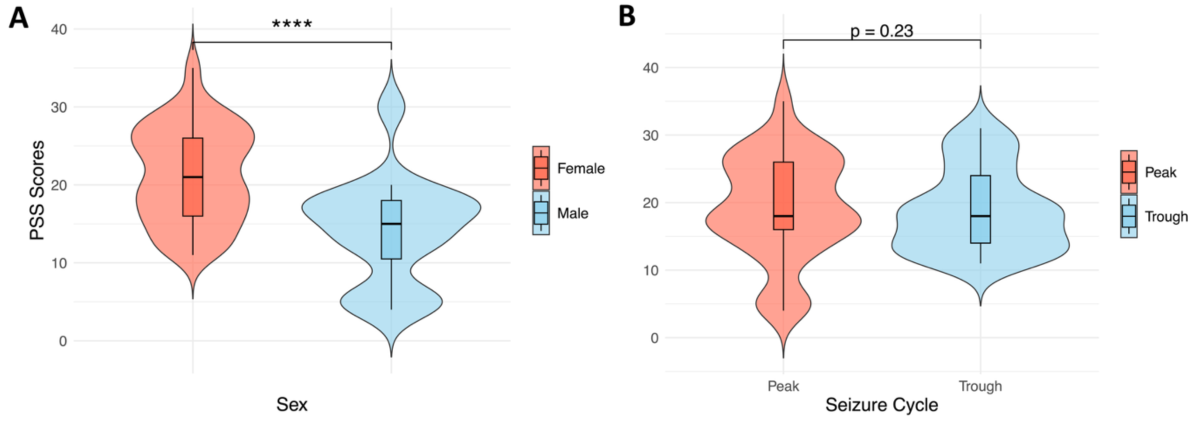

Supplementary Figure 9. The relationship between total Perceived Stress Scale scores and sex (A) and multiday seizure cycles (B) across the epilepsy cohort. Perceived Stress Scale (PSS) scores were derived using the PSS-10 questionnaire. (A) Red box plots represent PSS scores reported by females (n=36 observations), while blue box plots represent PSS scores reported by males (n=16 observations). (B) Red box plots represent PSS scores recorded during peaks in multiday seizure cycles (n=33 observations), and blue box plots represent PSS scores recorded during troughs (n=19 observations). Group differences were assessed using Mann–Whitney–Wilcoxon tests, with significant p-values ($p < 0.05$) indicated*. W-statistic [p-value] for the significant finding: Male vs Female = 4716 [$p<0.001$].

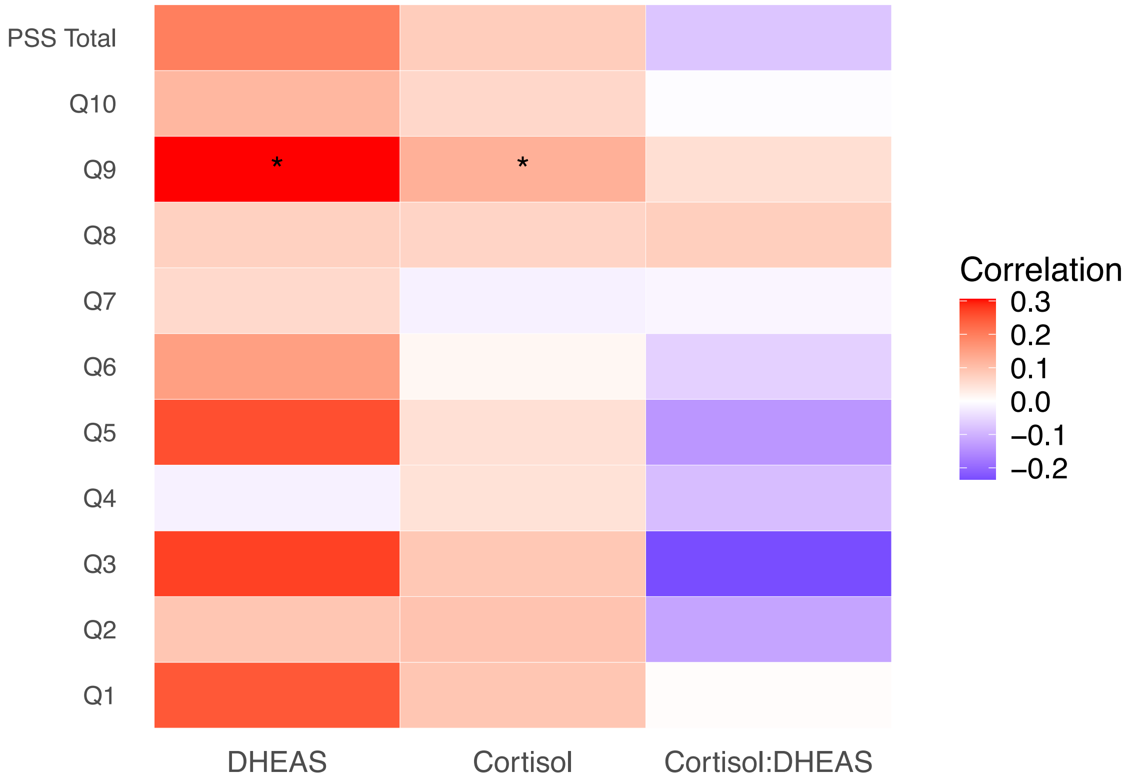

Supplementary Figure 10. Correlation matrix to assess relationships between individual items (questions Q1-10) on the Perceived Stress Scale (PSS) questionnaire and stress hormone concentrations (Dehydroepiandrosterone Sulphate (DHEAS), Cortisol and Cortisol:DHEAS ratio). DHEAS and Cortisol:DHEAS correlations used n=52 observations (one sample per participant per sampling period); Cortisol correlations used n=312 observations. * Indicates significant correlation ($p<0.05$) using Pearson's correlation coefficient. Pearson's r, t-statistic [p-value] for significant findings: DHEAS/Q9 = 0.30, t=2.26 [p=0.028]; Cortisol/Q9 = 0.12, t=2.20 [p=0.028].
